# Adipocyte-derived extracellular vesicles increase insulin secretion through transport of insulinotropic protein cargo

**DOI:** 10.1038/s41467-023-36148-1

**Published:** 2023-02-09

**Authors:** Konxhe Kulaj, Alexandra Harger, Michaela Bauer, Özüm S. Caliskan, Tilak Kumar Gupta, Dapi Menglin Chiang, Edward Milbank, Josefine Reber, Angelos Karlas, Petra Kotzbeck, David N. Sailer, Francesco Volta, Dominik Lutter, Sneha Prakash, Juliane Merl-Pham, Vasilis Ntziachristos, Hans Hauner, Michael W. Pfaffl, Matthias H. Tschöp, Timo D. Müller, Stefanie M. Hauck, Benjamin D. Engel, Jantje M. Gerdes, Paul T. Pfluger, Natalie Krahmer, Kerstin Stemmer

**Affiliations:** 1 Institute for Diabetes and Obesity, Helmholtz Zentrum München, Neuherberg, Germany; 2 German Center for Diabetes Research (DZD), Neuherberg, Germany; 3 Molecular Cell Biology, Institute for Theoretical Medicine, Medical Faculty, University of Augsburg, Augsburg, Germany; 4 Department of Molecular Structural Biology, Max Planck Institute of Biochemistry, Martinsried, Germany; 5 Division of Animal Physiology and Immunology, School of Life Sciences Weihenstephan, Technical University of Munich, Munich, Germany; 6 Institute of Biological and Medical Imaging, Helmholtz Zentrum München, Neuherberg, Germany; 7 Chair of Biological Imaging at the Central Institute for Translational Cancer Research (TranslaTUM), School of Medicine, Technical University of Munich, Munich, Germany; 8 Department for Vascular and Endovascular Surgery, Klinikum rechts der Isar, Technical University of Munich, Munich, Germany; 9 DZHK (German Centre for Cardiovascular Research), Partner Site Munich Heart Alliance, Munich, Germany; 10 Department of Surgery, Division of Plastic, Aesthetic and Reconstructive Surgery, Medical University of Graz, Graz, Austria; 11 Institute of Molecular Oncology and Functional Genomics, School of Medicine, Technical University of Munich, Munich, Germany; 12 Institute of Diabetes and Regeneration Research, Helmholtz Zentrum München, Neuherberg, Germany; 13 Department of Medicine and Surgery, University of Parma, Parma, Italy; 14 Metabolomics and Proteomics Core, Helmholtz Zentrum München, Neuherberg, Germany; 15 Institute for Nutritional Medicine, School of Medicine, Technical University of Munich, Munich, Germany; 16 ZIEL—Institute for Food & Health, Technical University of Munich, Freising, Germany; 17 Division of Metabolic Diseases, Department of Medicine, Technical University of Munich, Munich, Germany; 18 Helmholtz Pioneer Campus, Helmholtz Zentrum München, Neuherberg, Germany; 19 Biozentrum, University of Basel, Basel, Switzerland; 20 Research Unit NeuroBiology of Diabetes, Helmholtz Zentrum München, Neuherberg, Germany; 21 Chair of Neurobiology of Diabetes, TUM School of Medicine, Technical University of Munich, Munich, Germany

**Keywords:** Mechanisms of disease, Pre-diabetes

## Abstract

Adipocyte-derived extracellular vesicles (AdEVs) are membranous nanoparticles that convey communication from adipose tissue to other organs. Here, to delineate their role as messengers with glucoregulatory nature, we paired fluorescence AdEV-tracing and SILAC-labeling with (phospho)proteomics, and revealed that AdEVs transfer functional insulinotropic protein cargo into pancreatic β-cells. Upon transfer, AdEV proteins were subjects for phosphorylation, augmented insulinotropic GPCR/cAMP/PKA signaling by increasing total protein abundances and phosphosite dynamics, and ultimately enhanced 1st-phase glucose-stimulated insulin secretion (GSIS) in murine islets. Notably, insulinotropic effects were restricted to AdEVs isolated from obese and insulin resistant, but not lean mice, which was consistent with differential protein loads and AdEV luminal morphologies. Likewise, in vivo pre-treatment with AdEVs from obese but not lean mice amplified insulin secretion and glucose tolerance in mice. This data suggests that secreted AdEVs can inform pancreatic β-cells about insulin resistance in adipose tissue in order to amplify GSIS in times of increased insulin demand.

## Abstract (web summary)

Extracellular vesicles (EVs) convey inter-organ communication in health and disease. Here, the authors report that adipocyte-derived EVs isolated from insulin-resistant obese but not lean male mice stimulate insulin secretion via the targeted transfer of insulinotropic proteins from adipose tissue to β-cells.

## Introduction

Extracellular vesicles (EVs) constitute a heterogeneous group of lipid bilayer encased secreted vesicles, including endosome-originating exosomes (small EVs), plasma membrane-derived microvesicles (ectosomes)^1^, and a newly defined subgroup of mitochondria-derived vesicles^2,3^. By transferring bioactive proteins, lipids, RNAs and micro-RNAs (miRNAs) between cells and organs, EVs can reprogram cellular functions in paracrine or endocrine fashion under both homeostatic and pathological conditions^4^.

Recent findings suggest that white adipose tissue (WAT)-derived EVs play an important role in the onset of insulin resistance and glucose intolerance^5^. WAT-secreted EVs derive from adipocytes (AdEVs) or from cells of the stromal vascular fraction (SVF), including adipose tissue-resident macrophages (ATM-EVs) and represent an important constituent of the WAT secretome^6^. Both, AdEVs and ATM-EVs have been linked to the onset of insulin resistance in liver and muscle, which is likely mediated via the transfer of miRNAs such as miR-155 and miR-27a and modulation of PPARγ signaling in target tissues^5,7,8^. Impaired glucose tolerance may further result from AdEV-mediated changes in macrophage polarization in WAT^9,10^. In contrast to WAT EV-mediated effects in liver and muscle, effects of ATM-EVs and AdEVs in the pancreas are less clear. ATM-EVs isolated from obese mice decreased pancreatic insulin secretion and propagated β-cell proliferation by a miR-155 dependent mechanism in vivo and in vitro^11^. AdEVs from cytokine-treated adipocytes resulted in β-cell death and dysfunction, while EVs from 3T3-L1 adipocytes enhanced insulin secretion in vitro via an unknown mechanism^12^.

In this study, we aimed to fully delineate this mechanistic role of AdEVs as regulators of β-cell function in vivo and in vitro by focusing on a possible EV-mediated transfer of functional protein cargo from host to recipient cells. Following a detailed characterization of AdEVs from lean and diet-induced obese (DIO) mice using differential ultracentrifugation (dUC)- and size exclusion chromatography (SEC)-based isolation methods, we applied SILAC stable isotope in vitro and fluorescence AdEV labeling in vivo. This was paired with large-scale (phospho)proteome and miRNA profiling and functional assays to assess glucose-stimulated insulin secretion (GSIS) and glucose tolerance. We demonstrate that AdEVs transfer a functional insulinotropic protein cargo to pancreatic β-cells, which renders the cells more sensitive to a glucose stimulus and increases GSIS. AdEVs may therefore serve as signaling entity that amplifies insulin secretion independently from hyperglycemia. In addition, given the long-standing discussion regarding the quality of different AdEV isolation methods, we demonstrate conserved differences in lean vs. obese AdEV-encased protein cargo after both dUC and SEC.

## Results

### AdEVs from lean and obese mice differ in morphology

**Fig. 1:**
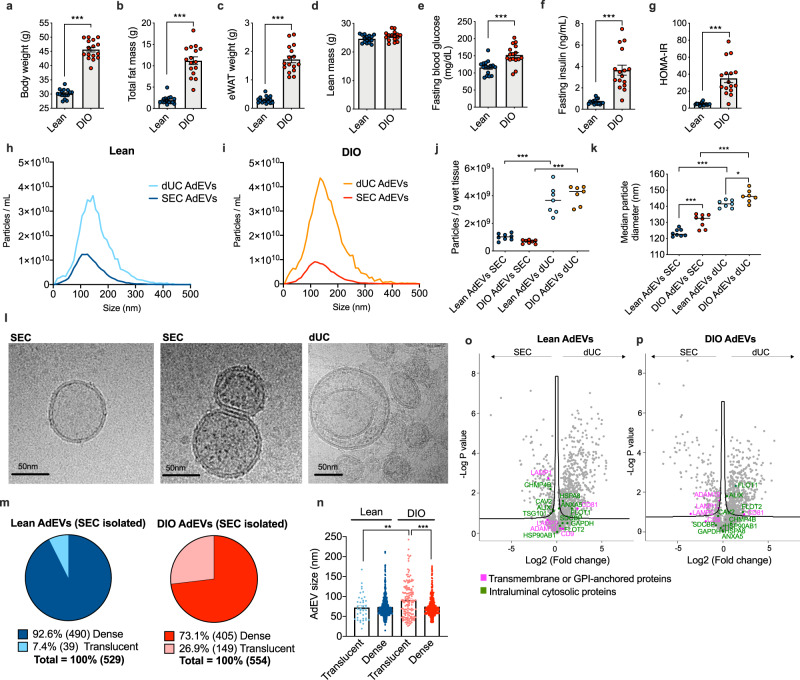
Comparative assessment of AdEVs isolated by dUC or SEC. Male lean and diet-induced obese (DIO) C57BL/6 J mice (*n* = 16 mice, 24–26 weeks of age, 16 weeks of feeding) were assessed for **a** body weight, **b** fat mass, **c** epididymal white adipose tissue (eWAT) mass, **d** lean mass, **e** fasting glucose, **f** fasting insulin, and **g** HOMA-IR values. **h**–**k** Nanoparticle Tracking Analysis (NTA) of adipocyte-derived extracellular vesicles (AdEVs, *n* = 7–8 biological replicates from two different EV isolation experiments) with size distributions of AdEVs from **h** lean and **i** DIO mice, **j** numbers of particles per gram wet tissue and **k** median particle diameters of the isolated EVs. **l** Representative cryo-TEM images from two independent AdEVs isolations using size exclusion chromatography (SEC) with translucent (left) and dense (middle) lumen or by differential ultracentrifugation (dUC) (right image). **m** Cryo-TEM-determined dense/translucent percentage, **n** median AdEV diameter of dense and translucent AdEVs. **o**–**p** LC-MS/MS analysis of the protein abundance of EV markers listed in the MISEV guideline. Comparison of EV markers in SEC- and dUC-isolated AdEVs from **o** lean and **p** DIO mice (*n* = 4 independent AdEV replicate samples. Each AdEV sample was isolated from visceral fat pads pooled from 5 DIO or 10 lean male 6–8 months old C57BL/6 J mice subjected to 4–6 months of HFD or chow). **a**–**p** Data are presented as mean values ± SEM, asterisks indicate *P < 0.05, **P < 0.01, ***P < 0.001. **a**–**g** Significance was determined by unpaired two-tailed t-tests, **j**, **k**, **n** One-Way ANOVA with Sidak’s post-test, or **o**, **p** One-Way ANOVA with a false discovery rate (FDR) of 0.1. Exact *P* values are (**a**–**b**, **f**, **g**, dietary effects) *P* < 0.0001, **e** *P* = 0.0003, (**j** isolation methods) *P* < 0.0001, **k** *P* < 0.0001 for all comparisons except for *P* = 0.0004 for lean AdEVs SEC vs. DIO AdEVs SEC and *P* = 0.390 for lean AdEVs dUC vs. DIO AdEVs dUC. Exact P values for EV size and lumina morphology are **n** *P* = 0.0011 (AdEV size of lean translucent vs. DIO translucent) and *P* < 0.0001 (AdEV size of DIO translucent vs. DIO dense).

AdEVs were isolated from mice fed standard chow diet (lean) or mice fed high fat diet (HFD) that later developed diet-induced obesity (DIO), with higher body weight (Fig. 1a), fat mass (Fig. 1b) and epididymal white adipose tissue (eWAT, Fig. 1c) and unchanged lean mass (Fig. 1d). Increased fasting glucose (Fig. 1e) and insulin (Fig. 1f) levels translated into higher HOMA-IR values in DIO mice (Fig. 1g), a measure for impaired insulin sensitivity. With these characteristics, DIO mice resemble key features of obesity and insulin resistance in humans^13,14^.

Using eWAT adipocyte fractions from lean and DIO mice, we applied dUC- and SEC-based isolation protocols to compare yields, purity and size distribution of the resulting vesicle populations (Fig. 1h–k). Under both dietary conditions, SEC resulted in narrower size distributions and lower particle yields compared to dUC (Fig. 1h, i). Per gram tissue, AdEV yields were comparable in lean and DIO mice (Fig. 1j). SEC-isolated AdEV populations had smaller median particle diameters compared to dUC-isolated AdEVs (Fig. 1k). Independent from the isolation method, AdEVs from DIO mice had significantly larger diameters compared to AdEVs from lean mice (Fig. 1k). Together, these findings demonstrate that EV isolation techniques and body composition are important determinants of AdEV morphology and composition.

Cryo-transmission electron microscopy (cryo-TEM) confirmed the characteristic membrane lipid bilayer structure of AdEVs (Fig. 1l, Supplementary Fig. 1). After analyzing 4733 dUC-derived AdEVs and 1083 SEC-derived AdEVs from a total of 697 cryo-TEM images, we found only 60 (1.3%) monolayer vesicles after dUC, and none after SEC (Supplementary Fig. 2a, b). Bilayer membranes had roughly double the width of monolayer membranes (Supplementary Fig. 2c). Notably, bilayer-encased lumina of AdEVs clearly differed in density; some AdEVs appeared electron translucent (Fig. 1l, left), whereas others appeared full of dense material (Fig. 1l, middle). We did not find visible organelle-like structures, such as mitochondria or parts thereof. The ratio of translucent vs. dense vesicles differed in SEC-derived AdEVs from lean vs. DIO mice. While 490 (92.6%) dense and 39 (7.4%) translucent AdEVs were isolated from lean mice, 405 (73.1%) dense and 149 (26.9%) translucent AdEVs were isolated from DIO mice (Fig. 1m). Dense AdEVs from lean and DIO mice were of similar size, translucent AdEVs from DIO were significantly larger (Fig. 1n). Accordingly, translucent vesicles may account for the observed size differences (Fig. 1k). AdEVs isolated with dUC showed similar dense and translucent lumina profiles, however, not all vesicles could be clearly categorized (Supplementary Fig. 2d–f).

LC-MS/MS-based proteome analyses of isolated AdEVs confirmed the abundance of typical EV markers in SEC- and dUC-derived AdEVs from lean or DIO mice (Fig. 1o, p; Source file). Consistent with the “minimal information for studies of extracellular vesicles” (MISEV) guideline^1^, these include transmembrane or GPI-anchored proteins and intraluminal cytosolic proteins. Abundances of single EV markers nonetheless differed between isolation methods and dietary conditions (Fig. 1o, p). In lean mice, SEC-derived AdEVs had significantly higher abundances of LAMP1 and CHMP4b, while dUC-derived AdEVs had higher abundances of CD81, FLOT1, HSPA8 and ANXA5. Whereas, AdEVs from DIO mice only showed a significant increase in FLOT1. Strikingly, one frequently described EV marker, CD63, was neither abundant in SEC- nor dUC-isolated AdEVs, which appear to resemble a previously described CD81- and CD9-positive, but CD63-negative EV population^15^. Moreover, the finding of CD63 positive EVs from human adipose tissue and human cultivated adipocytes (Supplementary Fig. 3a) points to significant species-specific differences in EV marker expression. Additional Western blot analyses revealed the presence of EV marker TSG101 in eWAT lysates and SEC-isolated AdEVs. The negative marker ribosomal protein RPL5 was present in WAT lysates but absent in AdEVs (Supplementary Fig. 3b). In addition, absence of the macrophage marker F4/80 in the eWAT adipocyte fraction after removal of the stromal vascular fraction (SVF) indicates no significant contamination of AdEVs with EVs from adipose tissue macrophages (Supplementary Fig. 3c).

### AdEV protein cargoes differ in lean and DIO mice and reflect the metabolic state of the tissue

**Fig. 2:**
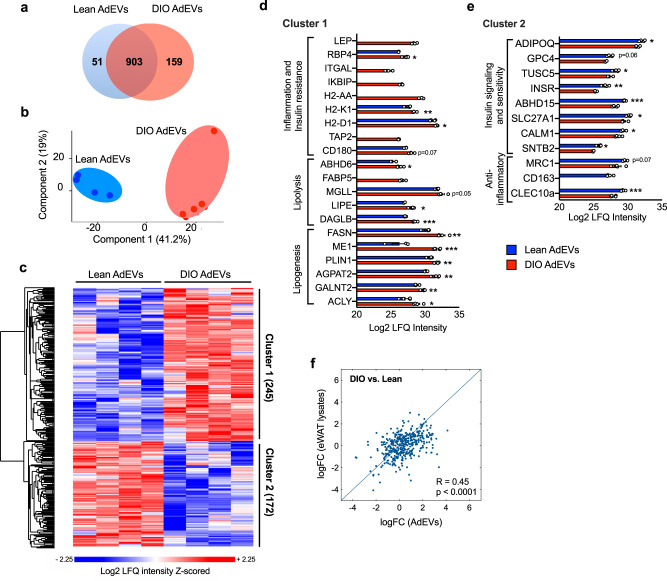
AdEV protein cargo in lean and DIO mice. AdEVs from lean and DIO mice (*n* = 4 independent AdEV replicate samples. Each AdEV sample was isolated from pooled visceral fat pads of 5 DIO or 10 lean male 6-8 months old C57BL/6 J mice subjected to 4-6 months of HFD or chow) were subjected to LC–MS/MS-based proteomics. **a** Venn Diagram and **b** principal component analysis (PCA) of proteins identified in lean and/or DIO AdEVs. **c** Heatmap of z-scored protein intensities for all uncovered proteins (ANOVA, FDR < 0.01), and **d**, **e** average Log2 LFQ intensities (±SEM) of selected proteins with different abundance in lean and DIO AdEVs. **d** Cluster 1: proteins involved in inflammation, insulin resistance, lipolysis and lipogenesis; **e** Cluster 2: proteins involved in anti-inflammatory responses, insulin signaling and sensitivity. **f** Correlation of log Fold Change (logFC) as derived from the difference (DIO vs. lean) in the expression levels of 371 eWAT lysates and AdEV proteins (Pearson correlation coefficient is 0.4520). **d**, **e** Data are presented as mean values ± SEM, asterisks indicate *P < 0.05, **P < 0.01, ***P < 0.001. Significance was determined by two-sample t-tests with a permutation-based FDR set to 0.1 **d**, **e**. Exact *P* values for Log2 LFQ Intensity differences between lean AdEVs vs DIO AdEVs are **d** *P* = 0.015165 (RBP4), *P* = 0.00761 (H2-K1), *P* = 0.0405 (H2-D1), *P* = 0.0747 (CD180), *P* = 0.0247 (ABHD6), *P* = 0.0545 (MGLL), *P* = 0.0261 (LIPE), *P* = 0.000101 (DAGLB), *P* = 0.00279 (FASN), *P* = 0.000471 (ME1), *P* = 0.00485 (PLIN1), *P* = 0.00239 (AGPAT2), *P* = 0.00352 (GALNT2), *P* = 0.0139 (ACLY) and **e** *P* = 0.0365 (ADIPOQ), *P* = 0.0657 (GPC4), *P* = 0.04802 (TUSC5), *P* = 0.0015 (INSR), *P* = 0.000719 (ABHD15), *P* = 0.0331 (SLC27A1), *P* = 0.0383 (CALM1), *P* = 0.0179 (SNTB2), *P* = 0.0713 (MRC1), *P* = 0.000958 (CLEC10a). The exact *P* value for correlation of log (Fold Change) of AdEVs vs. log (Fold Change) of eWAT lysates is *P* < 0.0001.

EVs are known to transfer host cell-specific cargoes^4^. The AdEV cargo from DIO mice should therefore reflect obesity-driven perturbations in lipid metabolism and insulin sensitivity reported for WAT^16^. Accordingly, we analyzed the protein cargoes of SEC-isolated AdEVs for differences in protein abundances between lean and DIO mice (Fig. 2, Source file). 51 out of 1113 detected proteins were only abundant in AdEVs of lean mice while 159 proteins were exclusive to DIO AdEVs (Fig. 2a). A principal component analysis (PCA) confirmed the effect of body adiposity on AdEV protein composition (Fig. 2b). In total, we observed 417 proteins with significantly different abundance in lean and DIO-derived AdEVs. 245 proteins were more abundant in DIO-derived AdEVs (Fig. 2c, cluster 1).

Cluster 1 included the obesity-related adipokines LEP^17^ and RBP4^18^ (Fig. 2d), proteins associated with inflammatory reactions to overnutrition (ITGAL, IKBIP), MHC class II members (H2-AA, H2-K1, H2-D1) and inflammatory TLR4 signaling members (TAP2 and CD180)^19–21^. As expected, AdEVs from DIO mice were enriched with proteins involved in lipolysis and fatty acid transport (ABHD6, FABP5, MGLL, LIPE, DAGLB), and enhanced lipogenesis and lipid droplet formation (FASN, ME1, PLIN1, AGPAT2, GALNT2, ACLY)^16^. The abundance of different obesity related proteins such as LEP, ITGAL, IKBIP, H2-AA, TAP2 and FABP5 was very low in AdEVs from lean mice and was detected in only one or no biological replicate.

A second cluster comprised 172 proteins with higher abundance in AdEVs from lean mice (Fig. 2c, cluster 2): ADIPOQ, GPC4 and TUSC5 (Fig. 2e) are negatively correlated to body weight and likely protective against insulin resistance; INSR, ABHD15, SLC27A1, CALM1, SNTB2) are mediators of insulin signaling, and MRC1, CD163, CLEC10a are anti-inflammatory^22–26^. Together, these findings suggest that AdEV protein signatures reflect the metabolic state of the parenting adipose tissues. Similar protein clustering in a parallel experiment where dUC was used to isolate AdEVs from lean vs. DIO mice supports this conclusion (Supplementary Fig. 4, Source file). Likewise, in another independent experiment we demonstrate a significant correlation between logarithmic fold change (DIO vs. lean) values of proteins derived from AdEVs or the parenting eWAT lysates (Fig. 2f).

### Uptake of AdEVs by β-cells increases insulinotropic protein abundance

**Fig. 3:**
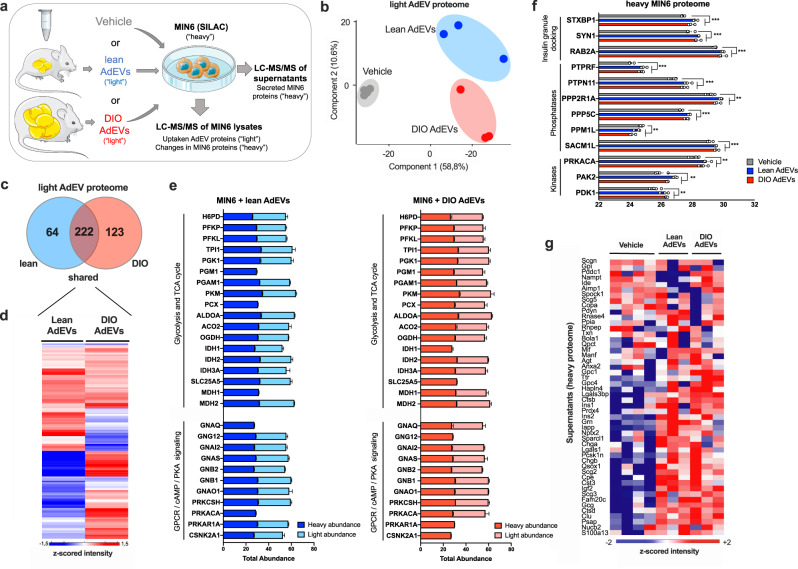
AdEV-mediated protein transfer into MIN6 cells. Proteomics of SILAC-labeled MIN6 cells treated with PBS (Vehicle) or AdEVs from lean or DIO mice (*n* = 3, biologically independent AdEV samples, each isolated from pooled visceral fat pads of 5 male C57BL/6 J DIO mice, 6–8 months of age subjected to 4–6 months of HFD or chow, *n* = 4 vehicle treated control samples). **a** Experimental setup. **b** PCA for “light” unlabeled proteins, and **c** Venn diagram representing the number of distinct vs. shared “light” unlabeled proteins transferred via lean or DIO AdEVs into MIN6 cells. **d** Heatmap for the median of z-scored protein intensities for all shared AdEV-derived “light” proteins in MIN6 cells following subtraction of the median intensities of the vehicle group (baseline subtraction). **e** Total abundances of “light” AdEV-derived proteins transferred into MIN6 cells via exposure to AdEVs from lean (light blue) or DIO (light red) mice (baseline-subtracted), or “heavy” proteins of the SILAC-treated host cells after treatment with lean (dark blue) or DIO (dark red) AdEVs. Proteins were clustering in different pathways, including glycolysis/TCA cycle and G-protein-coupled receptor (GPCR)/cyclic adenosine monophosphate (cAMP)/protein kinase A (PKA) signaling. **f** AdEV-induced changes of “heavy” cellular MIN6 proteins involved in insulin secretion. **g** Analysis of “heavy” MIN6 protein components of insulin granules secreted into the supernatant. **d**, **e** Data are presented as mean values ± SEM. Asterisks indicate *P < 0.05, **P < 0.01, ***P < 0.001. Significance was determined by two-sided t-tests with a permutation- based false discovery rate (FDR) of 0.1. Exact *P* values of AdEV treatment vs. vehicle are **f** *P* = 0.000348 (STXBP1), *P* = 0.001438 (SYN1), *P* = 0.00115 (RAB2A), *P* = 0.000102 (PTPRF), *P* = 0.000542 (PTPN11), *P* = 0.00616 (PPP2R1A), *P* = 0.000312 (PPP5C), *P* = 0.0133 (PPM1L), *P* = 0.000698 (SACM1L), *P* = 0.0105 (PRKACA), *P* = 0.00716 (PAK2) and *P* = 0.00353 (PDK1). The graphic in **a** was compiled using elements provided by Servier Medical Art (https://smart.servier.com).

Next, we applied stable isotope labeling (SILAC)^27^ of MIN6 cells to distinguish “heavy” ^13^C_6_^15^N_4_ arginine (Arg10) and ^13^C_6_^15^N_2_ lysine (Lys8)-labeled amino acids of β-cell proteins from unlabeled “light” (Arg6, Lys4) proteins originating from AdEVs (Fig. 3a).

Nearly complete “heavy” labeling efficiency (97%) was achieved after 8 passages of MIN6 cells in Arg10/Lys8 containing SILAC medium. Exposure of labeled MIN6 cells with vehicle, or unlabeled AdEVs from lean or DIO mice, lead to an enrichment of “light” proteins in MIN6 cells over the 3% background of unlabeled host cellular proteins, evidenced by a clear separation between groups in the PCA of unlabeled “light” proteins (Fig. 3b, Source file). After background subtraction to correct for unlabeled host cell proteins, we uncovered 409 AdEV-derived proteins, with 222 being shared between lean and DIO mice (Fig. 3c). 64 and 123 proteins were exclusively transferred by AdEVs from lean or DIO mice, respectively. Among those, many differed in their abundance (Fig. 3d). Comparative quantitative analyses between “light” AdEV-derived proteins and “heavy” equivalents from the host cells suggested that the delivery of AdEV protein cargo from lean and DIO mice can selectively enhance the total abundance of proteins involved in GSIS inducing pathways, such as such as glycolysis, TCA cycle and G-protein coupled receptor (GPCR)/cAMP/protein kinase A (PKA) signaling^28,29^ (Fig. 3e). While many proteins were transferred by AdEVs from lean and DIO mice, some were also transferred selectively. G-protein GNAQ, which is known to activate phospholipase C (PLC) and amplify insulin secretion^30^, and the catalytic subunit α of protein kinase A (PRKACA), a positive regulator of insulin secretion^31^, were only transferred by AdEVs from DIO mice. In contrast, CSNK2A1, a negative regulator of insulin secretion, was exclusively transferred by AdEVs from lean mice. We observed differences in proteins involved in glycolysis and the TCA cycle with PGM1, PCX and MDH1 exclusively transferred by DIO AdEVs, IDH1 and SLC25A5 by lean AdEVs (Fig. 3e). Next, we analyzed AdEV-induced changes of the heavy MIN6 proteome. Following 6hrs of exposure with AdEVs, we detected mild, diet-independent changes of the host cell proteome, with 388 MIN6 proteins significantly differing to those of vehicle-treated cells (two-sample student’s T-test with FDR = 0.1). These differences included higher abundances of STXBP1, SYN1 and RAB2A, which serve as crucial regulators of insulin granule docking (Fig. 3f). AdEV treatment further altered the abundance of phosphatases (PTPRF, PTPN11, PPP2R1A, PPP5C, PPM1L, SACM1L) and kinases (PRKACA, PAK2 and PDK1; Fig. 3f), suggesting an AdEV-mediated effect on cellular phospho-dynamics. This effect was independent of the isolation method, as AdEVs isolated with dUC caused similar responses in SILAC labeled MIN6 cells (Supplementary Fig. 5, Source file).

Finally, an additional analysis of secreted heavy MIN6 derived proteins in the cell culture supernatant revealed higher abundances of insulin granule components (Fig. 3g, Source file), supporting that AdEVs may play a functional role in stimulating basal insulin secretion.

### The AdEV protein cargo is a target for phosphorylation and amplifies glucose-stimulated phospho-dynamics in β-cells

**Fig. 4:**
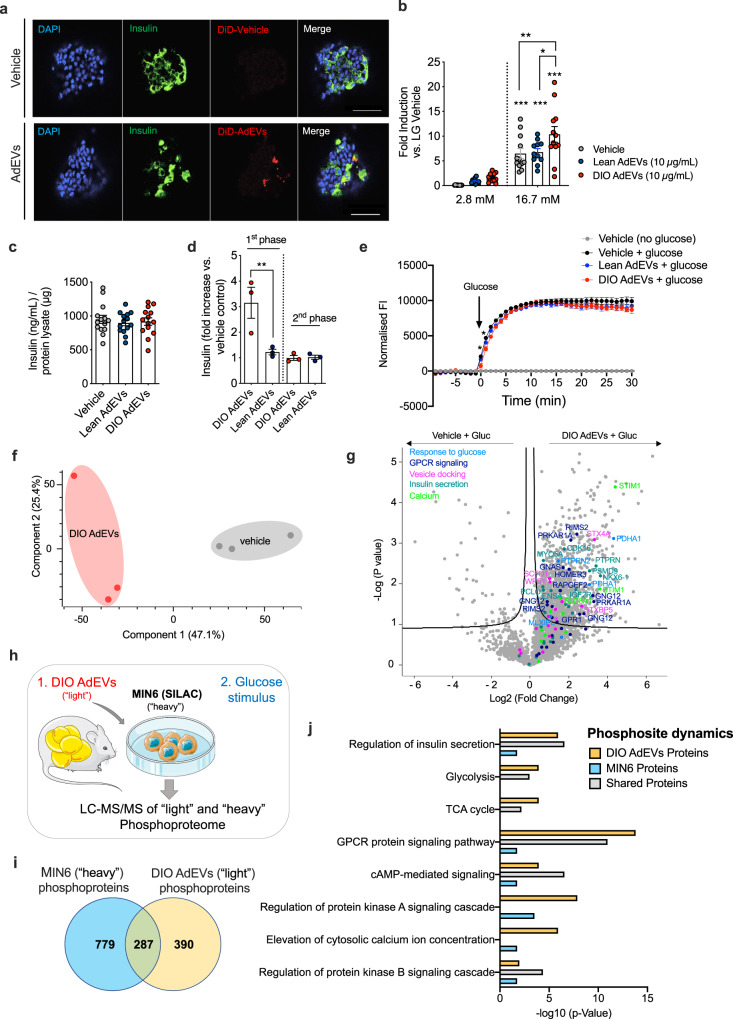
AdEVs from DIO mice enhance GSIS in pancreatic β-cells. Primary murine islets for experiments **a**–**d** were isolated from male C57BL/6 mice (16–18 weeks of age). AdEVs were isolated from male C57BL/6 J DIO mice (**a**–**j**: 4–6 months of HFD) or **b** from age and sex matched lean recipient C57BL/6 J mice. **a** Representative laser scanning confocal microscopy images of murine pancreatic islets after treatment with DiD-vehicle control (upper panel) or 10 µg of DiD-labeled AdEVs isolated from fat pads of DIO mice (lower panel). Scale bars: 50 µm. Nuclear staining via DAPI (blue), β-cell staining via anti-insulin antibody (green). **b** GSIS and **c** total insulin content in murine islets exposed to vehicle, lean or DIO AdEVs (10 µg/mL) followed by low glucose (LG) 2.8 mM and 16.7 mM glucose (*n* = 11–12 wells with primary murine islets treated with AdEVs from lean mice (*n* = 11 biologically independent samples), DIO mice (*n* = 12 biologically independent samples) from three independent isolations) or vehicle *n* = 12. **d** Dynamic islet perfusion assay in murine islets. Box plots represent the area under the curve of the first- and second phase of insulin secretion, expressed as fold increase vs. vehicle control (*n* = 3 biologically independent samples). **e** Real-time monitoring of glucose uptake into MIN6 cells expressing glucose sensitive Green Glifon600 and pretreated for 6 hrs with vehicle or AdEVs from lean or DIO mice (*n* = 4 biologically independent samples). Data represent baseline corrected fluorescence intensities (FI). **f** PCA and **g** Volcano Plot (ANOVA, FDR = 0.1) of MIN6 phosphoproteomes 15 min after switching from low to high glucose. Cells were pretreated with DIO AdEVs or vehicle for 6 hrs. **h**–**j** Independent replication experiment with SILAC-labeled and DIO AdEV-pretreated MIN6 cells. **h** experimental setup, **i** Venn diagram of significant phosphosite changes for heavy MIN6, light AdEV-derived proteins, or both after a glucose stimulus (FDR = 0.05). **j** Bar diagram of enrichment analysis (two-sided Fisher’s Exact Test, FDR = 0.005) of MIN6 and AdEV-specific phosphosite changes related to pathways involved in insulin secretion. Significance levels for the different enrichment classes are displayed as -log10 p-values. **b**–**e** Data are presented as mean values ± SEM. Asterisks indicate *P < 0.05, **P < 0.01, ***P < 0.001. Significance was determined by **b** two-way ANOVA and Tukey’s multiple comparisons test or **d** ordinary one-way ANOVA and Sidak’s multiple comparisons test. Exact *P* values are **b** *P* = 0.0047 for DIO AdEV treatment vs. vehicle at the 16.7 mM glucose condition; **c** *P* = 0.011 for the comparison of lean AdEV vs. DIO AdEV treatment and **d** *P* = 0.0025. **e** For exact *p*-values at individual time points see the data source files. The graphic in **h** was compiled using elements provided by Servier Medical Art (https://smart.servier.com).

To functionally assess the insulinotropic role of AdEVs under basal and glucose stimulated conditions, we next conducted insulin secretion studies in freshly isolated murine pancreatic islets. First, we confirmed the enrichment of fluorescence (DiD)-labeled AdEVs in β-cells of primary murine islets, 4 hrs post exposure (Fig. 4a). In murine islets pre-treated for 6 hrs with AdEVs from lean and DIO mice under low-glucose conditions (2.8 mM), we observed a mild but non-significant increase in insulin secretion (Fig. 4b). After switching to high glucose (16.7 mM), GSIS was significantly elevated in islets pre-treated with AdEVs from DIO mice (*P* = 0.0047 vs. vehicle) but not from lean mice (*P* = 0.97 vs. vehicle, Fig. 4b). AdEV pre-treatment did not affect cellular insulin content, suggesting an augmented release of preformed granules (Fig. 4c). Direct insulinotropic actions of AdEVs from DIO but not lean mice on the 1^st^ phase of GSIS were next corroborated in murine islets subjected to a dynamic islet perfusion assay (Fig. 4d). Pre-treatment with AdEVs from DIO but not from lean mice slightly blunted glucose uptake, however only within the first 2 min after glucose addition (Fig. 4e), suggesting that DIO AdEVs enhance the capacity of β-cells to translate a comparable glucose uptake into a higher insulin secretion.

First phase insulin secretion is a rapid process and strongly relies on the modulation of protein activities by post-translational modifications such as protein phosphorylation, rather than on altered gene or protein expression^32^. Hypothesizing that elevated GSIS in MIN6 cells pre-treated with DIO AdEVs should be associated with distinctly altered phosphorylation patterns of GSIS-related proteins, we next conducted phosphoproteome analysis of MIN6 cells incubated for 6 hrs (low glucose) with vehicle or AdEVs followed by a 15 min high-glucose bolus (Source file). The requirement of high protein amounts for phosphoproteome analyses restricted our studies to SEC-isolated AdEVs from DIO mice. The glucose bolus induced a strong phosphorylation response on the MIN6 proteome that was significantly exacerbated when MIN6 cells were pretreated with DIO AdEVs (Fig. 4f). This response included altered phosphorylation patterns for proteins directly involved in mediating the response to glucose (MLXIPL, PTPRN2, PDHA1), as well as G-protein signaling (GNAS, GNG12, GPR1, HOMER3, RAPGEF2, RIMS2, PRKAR1A), activation of calcium channels (STIM1, SLC8A1), the docking of insulin granules (STX4A, STXBP5, WFS1) and insulin secretion (IGFRR, PCLO, CDK16, ENSA, ARPP19, NKX6.1, MYO5A) (Fig. 4g).

To elucidate if DIO AdEV-derived proteins are (1) direct substrates of kinases and phosphatases in the MIN6 cells and (2) actively contribute to the overall glucose stimulated phosphosite-dynamics, we next performed phosphoproteome analysis in SILAC labeled MIN6 cells (Fig. 4h, Source file) by pre-treating MIN6 cells for 6 hrs with vehicle or DIO AdEVs prior to a glucose stimulus. For the comparative analysis of the “light” DIO AdEV phosphoproteome at low and high glucose conditions, we first performed a background subtraction in order to correct for possible unlabeled host cell phosphoproteins. Stimulation with glucose significantly increased the signal intensities for 677 phosphosites on “light” DIO AdEV-derived proteins and 1066 phosphosites on “heavy” MIN6 proteins (FDR = 0.05), with an overlap of 287 phosphosites (Fig. 4i). Enrichment analyses (FDR = 0.05) of either shared, MIN6- or DIO AdEV-specific phosphorylation changes suggested the concerted induction of pathways involved in insulin secretion, including GPCR/cAMP/PKA signaling and calcium elevations in the cytosol (Fig. 4j). These findings demonstrate that DIO AdEVs are direct targets for phosphorylation or dephosphorylation and these post-translational modifications contribute to the overall phosphosite dynamics of MIN6 cells.

### Functional contribution of AdEV miRNA cargo to GSIS in islets

In addition to protein cargo, AdEVs can transfer miRNAs, which may further contribute to the insulinotropic signaling in pancreatic β-cells. When analyzing AdEVs from lean or DIO mice for the presence of encased miRNAs (Supplementary Methods), we found 139 out of a pre-defined panel of 372 miRNAs. Two-way hierarchical clustering revealed a total of 50 miRNAs, which were differentially represented in AdEVs derived from lean vs. DIO mice (Supplementary Fig. 6a). Of those, 19 miRNAs were significantly different between the groups (Supplementary Table 1). Notably, a total of 22 miRNAs (with 8 miRNAs significantly different between lean and DIO-derived AdEVs) had been linked with GSIS and insulin gene regulation (Supplementary Table 2). In majority these miRNAs are negative regulators of insulin secretion and are significantly diminished in DIO compared to lean AdEVs. To study their functional relevance, AdEVs from DIO mice were transfected with a pool of four miRNA mimics for miR-29a-3p, miR-200a-3p, miR-218-5p and miR-322-5p and subjected to murine islets. Under low-glucose conditions, neither vehicle nor AdEVs with control miRNA or the miRNA mimic pool had an influence on insulin secretion (Supplementary Fig. 6b). Under high glucose conditions, we found a temporary first-phase GSIS in islets exposed to vehicle or AdEVs with control miRNA, but not in islets exposed to AdEVs with miRNA mimics. Fluorescence labeled non-EV encapsulated siRNAs were not incorporated into pancreatic islets (Supplementary Fig. 6c, d), demonstrating that AdEVs were required for the miRNA transfer into islets. This data indicates that a selective depletion of inhibitory miRNAs in DIO AdEVs could contribute to an overall increase in GSIS. Whether such effects are compensated by higher DIO AdEV secretion rates due to higher body adiposity remains to be tested.

### Pancreatic enrichment of DiR labeled AdEVs in vivo

**Fig. 5:**
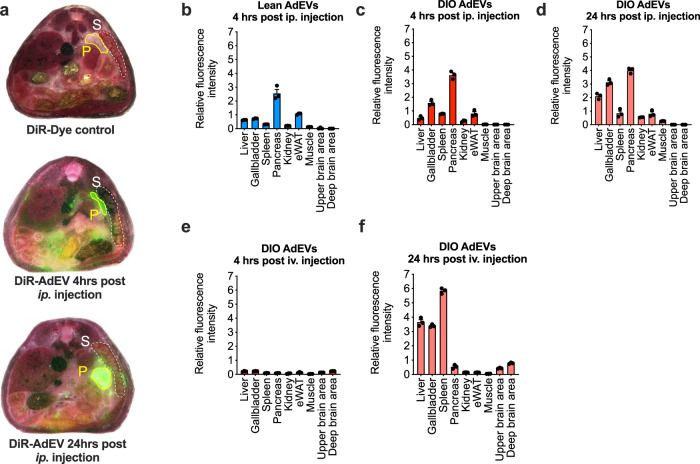
Biodistribution of AdEVs in mice. **a** Representative cross-sections of multiscale and multispectral images of whole cryo-sliced male lean C57BL/6 J mice (11 to 16 weeks of age) after ip. injection with DiR vehicle control (upper panels) or DiR-labeled AdEVs DIO mice (middle and lower panel). Cross-sections show the pancreas (P) and spleen (S). **b**–**f** Relative quantifications of fluorescence intensities in target organs of the recipient mice treated with DiR-labeled AdEVs isolated from **b** lean or **c**–**f** DIO male C57BL/6 J mice, **b**, **c** 4 hrs or **d** 24 hrs, after intraperitoneal (ip.) injection, as well as **e** 4 hrs, or **f** 24 hrs after intravenous iv. injection. In each experimental setup, three representative slices from *n* = 2 independently AdEV-injected mice were analyzed. Data are presented as mean values ± SEM.

To study in vivo relevance, we addressed the biodistribution kinetics of AdEVs in mice. Fluorescence (DiR) labeled AdEVs were either injected intraperitoneally (ip.) or intravenously (iv.) into lean C57BL/6 J mice, which were subsequently subjected to whole mouse cryo-slicing coupled to automated fluorescence detection. Consistent with a previous report^33^, we observed no dye accumulation in DiR injected control mice (Fig. 5a, Supplementary Fig. 7), while the DiR-labeled AdEVs accumulated in several target organs, depending on the timing and the route of injection (Fig. 5b–f, Supplementary Fig. 7). Four hours post ip. injection, DiR-labeled AdEVs accumulated in seven organs, with highest intensities found in the pancreas, followed by the gallbladder and eWAT, spleen, liver, kidney and skeletal muscle. Distribution patterns were similar for AdEVs from lean (Fig. 5b) and DIO mice (Fig. 5c). Fluorescence intensities remained highest in pancreata even 24 hrs after ip. injection of DiR-labeled AdEVs (Fig. 5d). Intravenous injection resulted in a delayed organ accumulation of AdEVs, with no obvious signal 4 hrs post injection (Fig. 5e). After 24 hrs after injection we found highest signals in spleen, liver and gallbladder followed by an enrichment in the pancreas, and a mild additional signal in upper and deeper brain areas (Fig. 5f).

### Pancreatic enrichment of AdEVs increases GSIS and improves glycemic control in mice

**Fig. 6:**
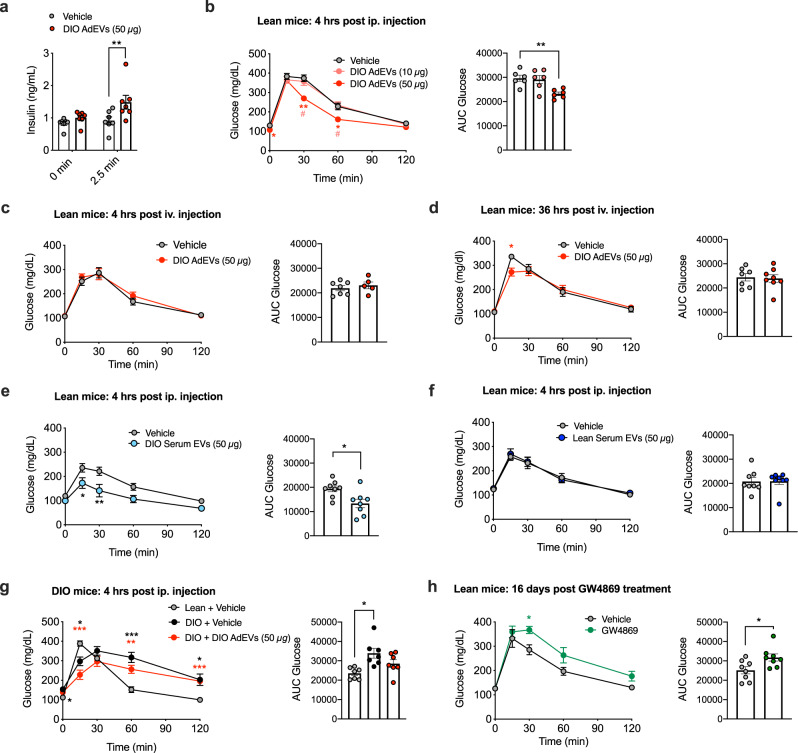
Glucoregulatory effects of AdEVs in mice. DIO AdEVs were isolated from male C57BL/6 J DIO mice subjected to HFD for 4–6 months **a**–**d**, **g**. All recipient mice **a**–**g** were male C57BL/6 J mice. **a** Insulin secretion in mice (12 weeks of age, *n* = 7 mice) at baseline and 2.5 min after glucose stimulation following a 4 hrs pretreatment with vehicle or 50 μg DIO AdEVs. **b**–**h** Glucose excursions (left panels) and corresponding area under curve (AUC, right panels) values following an **b** ipGTT in lean mice, 4 hrs after ip. pretreatment with vehicle vs. 10 or 50 μg of DIO AdEVs (*n* = 6 mice, 12 weeks of age), **c**, **d** ipGTT in lean mice, 4 hrs (**c**: *n* = 7 vehicle treated mice, *n* = 5 AdEV treated mice) or 36 hrs (**d**: *n* = 7 vehicle treated mice, *n* = 8 AdEV treated mice) after iv. pretreatment with vehicle vs. 50 μg of DIO AdEVs. **e**, **f** Glucose excursions and AUCs in lean mice 4 hrs after pre-treatment with vehicle or 50 μg of serum EVs isolated from 4-6 months old male C57BL/6 J DIO **e** or lean mice **f** (*n* = 8 mice in both experiments). **g** ipGTT in DIO recipient mice (3 months HFD, average body weight 45.5 g) and age-matched lean mice (average body weight 32.1 g), 4 hrs after ip. pretreatment with vehicle vs. 50 μg of DIO AdEVs (*n* = 7 mice). **h** ipGTT in lean 30-weeks-old male C57BL/6 J mice injected daily for 16 days with the small molecule exosome secretion inhibitor GW4869 (1 mg/kg), or vehicle (5% DMSO in 0.9% NaCl), *n* = 8 mice. **d**, **e **Data are presented as mean values ± SEM. Asterisks indicate *P < 0.05, **P < 0.01, ***P < 0.001. Significance was determined by two-way ANOVA and Sidak’s multiple comparisons test or unpaired two-sided t-test for AUC values. Exact *P* values of treatment vs. control are **a** adjusted *P* = 0.01, **b** *P* = 0.0247 (vehicle vs. 50 μg DIO AdEVs, 0 min), *P* = 0.002 (vehicle vs. 50 μg DIO AdEVs, 30 min), *P* = 0.0144 (10 μg DIO AdEVs vs. 50 μg DIO AdEVs, 30 min), *P* = 0.0289 (vehicle vs. 50 μg DIO AdEVs, 60 min), *P* = 0.0193 (10 μg DIO AdEVs vs. 50 μg DIO AdEVs, 60 min) and *P* = 0.0071 (for AUC Glucose), **d** *P* = 0.0275, **e** *P* = 0.0272 (15 min), *P* = 0.0025 (30 min) and *P* = 0.0127 (for AUC Glucose), **g** *P* = 0.0348 (lean + vehicle vs. DIO + vehicle, 0 min), *P* = 0.0143 (lean + vehicle vs. DIO + vehicle, 15 min), *P* = 0.0005 (lean + vehicle vs. DIO + 50 μg DIO AdEVs, 15 min), *P* = 0.0009 (lean + vehicle vs. DIO + vehicle, 60 min), *P* = 0.0043 (lean + vehicle vs. DIO + 50 μg DIO AdEVs, 60 min), *P* = 0.0271 (lean + vehicle vs. DIO + vehicle, 120 min), *P* = 0.0001 (lean + vehicle vs. DIO + 50 μg DIO AdEVs, 120 min) and *P* = 0.017 (for AUC Glucose), **h** *P* = 0.032 and *P* = 0.0238 (for AUC Glucose).

Next, prompted by the pancreatic accumulation of labeled AdEV, we assessed how a comparable pre-injection (4 hrs, ip.) with vehicle or DIO AdEVs could affect glucose-stimulated insulin secretion and gluco-regulation in vivo in lean mice. Under basal conditions, pre-treatment with 50 µg DIO AdEVs caused a mild but non-significant increase of insulin secretion before (0 min) and a significantly increased insulin secretion 2.5 min after the glucose bolus, compared to vehicle controls (Fig. 6a). Glucose tolerance was improved by 50 µg but not 10 µg DIO AdEVs, suggesting a dose dependency (Fig. 6b).

Consistent with the time-delayed accumulation of iv. injected AdEVs in the pancreas (Fig. 5f), improvements of glucose tolerance only occurred 36 hrs but not 4 hrs post iv. injection of DIO AdEVs in another set of lean mice, compared to vehicle treated controls (Fig. 6c, d). Serum EVs (50 µg) from DIO mice, which contain a mixture of different organ-derived EVs including detectable amounts of AdEVs^34^, elicited a comparable improvement in glucose tolerance in recipient mice (Fig. 6e). Interestingly and consistent with the findings from isolated pancreatic islets (Fig. 4e), glucose excursions remained unchanged when mice were treated with serum EVs from lean mice, compared to vehicle-treated controls (Fig. 6f). This data supports that DIO AdEV pretreatment provides glucoregulatory benefits in lean recipient mice.

To assess whether circulating obesogenic and inflammatory factors such as hormones, adipocytokines or lipid species that are prevalent in DIO mice could mask any AdEV benefits on glucose tolerance, we repeated our DIO AdEV ip. pretreatment studies in DIO mice. Acute ip. injections of DIO AdEVs 4 hrs prior a GTT were able to blunt, yet not fully revert the impaired glucose tolerance of vehicle-treated DIO mice, compared to lean controls (Fig. 6g). Last, lean C57BL/6 J mice were injected daily for 16 days with GW4869 (1 mg/kg), a noncompetitive inhibitor of sphingomyelinase (SMase) that blocks the generation and release of exosomes^35^. GW4869 treatment induced a mild impairment in glucose tolerance in lean mice (Fig. 6h), but had no effects on body weight, food intake or energy expenditure (Supplementary Fig. 8), evidencing a role of EVs in glucose metabolism.

### AdEVs but not the corresponding EVs from the SVF of human liposuction patients are enriched with proteins involved in insulin secretion

**Fig. 7:**
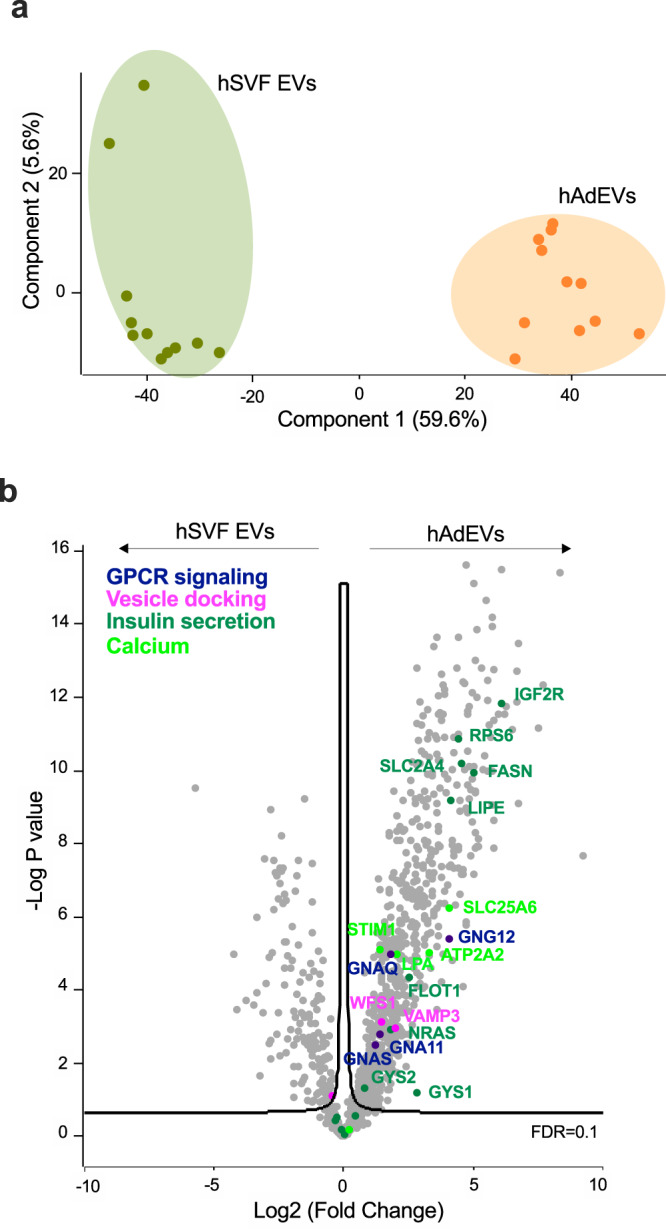
Characterization of human AdEVs and stromal vesicular fraction (SVF) derived EV proteins. EVs isolated from the stromal vascular fraction (SVF) and adipocyte fraction of liposuction samples from 11 self-reported female human subjects were analyzed by LC-MS/MS **a** PCA space differences and **b** Volcano plot displaying the significantly enriched expression of proteins involved in GPCR and calcium signaling, vesicle docking and insulin secretion in human adipocyte EVs (hAdEVs) compared to their expression in human SVF EVs. Significance was determined by two-samples t-test with 0.1 FDR.

Our data suggest that AdEVs from obese and insulin resistant mice increase insulin secretion. In contrast, ATM-EVs from obese adipose tissue of mice were reported to decrease pancreatic insulin secretion^11^. This suggests a mechanism whereby increasing adipose tissue inflammation and increased ATM-EVs can counteract insulinotropic effects of AdEVs. Accordingly, ATM-EVs should lack the enrichment of proteins promoting insulin secretion. To prove this and to provide human relevance, we isolated and compared the proteome profiles of EVs isolated from the adipocyte fraction and the ATM-containing SVF of human liposuction samples (Supplementary Table 3, Supplementary Methods). Protein fractions from hSVF-EVs and hAdEVs were clearly separated in the PCA space (Fig. 7a, Source file). hAdEVs but not hSVF-EVs were significantly enriched with proteins involved in GPCR and calcium signaling, vesicle docking and insulin secretion (Fig. 7b), which is consistent with our murine data that suggest a direct role for AdEVs in pancreatic GSIS.

## Discussion

Our study provides evidence that AdEVs serve as a signaling entity that can amplify insulin secretion in pancreatic β-cells. Such insulinotropic action appears to depend on the metabolic state of the adipose tissue, with AdEVs from DIO mice transferring a functional insulinotropic protein cargo that renders β-cells more sensitive to a glucose stimulus, compared to AdEVs isolated from lean mice. Overall, our data suggests that AdEVs from obese and insulin resistant adipocytes can promote enhanced compensatory insulin secretion often observed in the early stages of type 2 diabetes (T2D)^36^.

Our model is supported by the following: (1) AdEVs isolated from lean and obese adipose tissue differ in their insulinotropic protein and miRNA cargo, thereby are able to contribute to different secretomes. Although AdEV secretion kinetics from WAT exposed to metabolic stressors remain to be studied in greater detail, it appears likely that larger fat depots under obese conditions secrete more AdEVs^34,37^. Accordingly, in lean mice the relative amount of AdEVs in proportion to other EVs will likely be low, compared to a higher relative proportion of circulating DIO AdEVs in DIO mice. Whether morphologic differences in size and density observed for lean vs. DIO AdEVs contribute to proportional variations remains to be tested. (2) Using fluorescence labeling, we demonstrate that AdEVs, independent of the route of injection (ip. versus iv.), accumulate in the pancreas, albeit with slightly differing biokinetic profiles. We show that ip. injection leads to a faster and greater uptake into the pancreas compared to the iv. injection. A similar delay in pancreatic uptake of EVs had been reported for HEK293T-derived EVs^38^. Both ip. and iv. injected AdEVs exert comparable in vivo benefits on glucose tolerance. In addition, 4 hrs post ip. injection of AdEVs we observed a significant increase of proteins involved in insulin secretion. (3) We suggest that DIO AdEV protein cargo is a major functional driver of insulin secretion. SILAC-based proteome analyses allowed us to show that exposure to DIO AdEVs increases the total and relative abundance of distinct proteins involved in the metabolic amplification of GSIS^28^ such as GL/FFA cycling and GPCR/cAMP signaling. The latter also includes the transfer of mitochondrial proteins (such as IDH2, IDH3, OGDH, ACO2, PCX) by AdEVs, confirming previous findings by Crewe and co-workers^39^. Of note, our LC-MS data were not confirmed by the presence of visible mitochondrial structures in our cryo-TEM-based inspection of AdEV lumina. This may suggest the additional presence of either a distinct sub-species of shed mitochondrial vesicles, or EVs stemming from amphisome formation^40^. Moreover, we demonstrate that AdEV proteins tightly linked to insulin secretion are phosphorylated inside the β-cells, suggesting that AdEV-derived proteins actively participate in cellular signaling events. Together, these findings suggest that the transfer of distinct proteins by AdEVs can enhance metabolic pathways involved in insulin secretion by a transcriptionally- and translationally-independent mechanism. (4) In AdEVs from lean and DIO mice, we found miRNAs previously described as repressors of insulin secretion. Reconstituting DIO AdEVs with the respective miRNA mimics blunted their insulinotropic response. Accordingly, the lower abundance of “repressors” in AdEVs from DIO mice may translate into increased GSIS in islets and mice. However, the relative lack of inhibitory miRNAs may be counterbalanced by overall greater DIO AdEV secretion rates^34,41^, thus questioning whether it is the miRNA cargo or differing secretion rates that primarily contribute to variations in insulinotropic effects of DIO AdEVs. (5) Last, we demonstrate that the insulinotropic protein cargo is conserved in human adipose tissue and is only present in the adipocyte derived AdEVs, not in EVs derived from other cell types present in the SVF, such as ATMs.

In summary, our study corroborates and expands the concept of adipose tissue EVs as mediators for trans-organ communication^7,39,42^. Furthermore, our study is consistent with previous in vitro findings showing an enhanced insulin secretion in human Ins1β cells after treatment with EVs from 3T3-L1 adipocytes^12^. Notably, AdEVs isolated from cytokine-treated adipocytes^12^, a model for adipose tissue inflammation, or EVs from murine ATMs^11^ had previously been linked with β-cell death and dysfunction. Our data is not in conflict with these findings, but rather extends on those earlier reports. Specifically, we suggest a model whereby AdEVs from insulin resistant DIO mice augment insulin secretion, thus facilitating euglycemia in an early state of increased insulin demand. Following disease progression and increasing AT inflammation, such beneficial contributions from AdEVs to glucose metabolism may be gradually displaced by detrimental effects of EVs secreted from ATMs or increasingly inflamed adipocytes. Future studies should aim at exploring and delineating this acute vs. chronic impact of AdEVs on various stages of T2D progression. Likewise, such studies should test whether the therapeutic blockage of ATM EVs and/or the generation of artificial AdEVs mimetics with insulinotropic cargo can serve as a treatment strategy to enhance insulin secretion in patients with T2D.

## Methods

### Ethics declarations

The animal experiments were performed in accordance with the European guidelines under permission of the local Animal Ethics Committee of the state of Bavaria, Germany. Human adipose tissue donors (all female, no financial compensations) gave written informed consent. The study protocol was approved by the ethics committee of the Technical University of Munich.

### AdEV isolation from murine and human WAT

Male C57BL/6 J mice were purchased from Janvier Labs at an age of 6-8 weeks. Upon arrival, mice were group-housed and kept in a constant environment with the ambient temperature set to 22 ± 2 °C with constant humidity (45–65%) and a 12 hrs/12 hrs light/dark cycle. Murine AdEVs were isolated from the adipocyte fraction of the epididymal white adipose tissue (eWAT) of male C57BL/6 J mice fed with high fat diet (HFD, #D12332, Research Diets Inc) or chow (Altromin #1310) for 4–6 months. Murine eWAT processing and subsequent AdEV isolations by SEC or dUC, as well as the isolation of AdEVs and SVF-EVs from human liposuction samples are described in the supplementary information.

### EV protein quantification and characterization

Total EV protein concentrations were determined by BCA assays (Thermo Fisher Scientific). Nano Tracking Analyses (NTA) were performed with a ZetaView® PMX 110 (Particle Metrix GmbH). CryoTEM was conducted using fresh EV samples (4 μL) and a Tecnai G2 Polara transmission electron microscope (FEI, Thermo Fisher Scientific) equipped with a field emission gun operated at 300 kV, a post-column energy filter (Gatan), and a 3838 × 3710 Gatan K2 Summit direct detection camera operated in counting mode. Further details on all techniques are given in the supplementary methods.

### LC-MS/MS analysis

Cells or AdEVs were lysed in SDC buffer (2% (w/v) SDC for proteomics and 4% (w/v) SDC for phosphoproteomics, 100 mM Tris–HCl pH8.5) and peptides were digested and purified according to^43^. Samples for phosphoproteome analyses were prepared by following the EasyPhos workflow^44^. Human EVs from the SVF and adipocyte fraction of liposuction patients were subjected to tryptic digestion using a modified filter aided sample preparation (FASP) procedure^45^. Specific details on sample preparation LC-MS/MS analysis are given in the supplementary material.

### SILAC labeling and (phospho)proteomics

Stable isotope labeling by amino acids in cell culture (SILAC) was achieved by 8 passages of MIN6 cells cultured in high glucose DMEM-SILAC media supplemented with dialyzed FCS (15%), penicillin & streptomycin (100 units/mL), β-mecaptoethanol (50 μM) and the two “heavy” amino acids (Arg10) and (Lys8). Details on the subsequent LC-MS/MS analyses are given in the supplementary material. Total proteomes were measured for human liposuction samples and for MIN6 cells treated with PBS or AdEVs from lean and obese mice, phosphoproteome analyses were conducted for MIN6 cells treated with PBS or AdEVs from DIO mice following glucose stimulation.

### Cellular glucose uptake, islet preparation and GSIS

Glucose uptake was determined in AdEV pre-treated Green Glifon600-expressing MIN6 cells as described in the supplementary methods. Islets of Langerhans were isolated from the murine pancreata, maintained and hand-picked for further experiments as described in the supplementary information and before^46^. Hand-picked islets (50 per condition) were incubated for 6 hrs with AdEVs from lean and DIO mice or vehicle using culture medium previously depleted from FBS-derived EVs by overnight centrifugation at 100,000 g and 4 °C. Islets were then transferred to a 96-well V-bottom plate (5 per well), washed and pre-incubated for 1 hr in KRB containing 0.1% BSA and 2 mM glucose, then subjected to KRB containing 2.8 mM glucose for 1 hr (low glucose) followed by a 1 hr incubation period in KRB containing 16.7 mM glucose (high glucose). Insulin contents of supernatants were determined by ELISA (Crystral Chem Inc). For normalization, islets were lysed in RIPA buffer supplemented with protease inhibitor and total insulin and protein contents were measured by ELISA and BCA assays, respectively.

### Fluorescent AdEV uptake in vivo and in vitro

For ex vivo and in vivo tracking studies, we fluorescence-labeled AdEVs with DiR for assessing the systemic biodistribution, or with CellBrite™ Red Cytoplasmatic Membrane DiD dye (Biotium) to study AdEV uptake into β-cells. Labeled AdEVs were either injected intraperitoneally or intravenously into 12- to 16-week-old male C57BL/6 J mice, or subjected to freshly isolated murine pancreatic islets. Details are given in the supplementary material.

### Glucose tolerance test (GTT) and GSIS in AdEV-treated mice

Chow-fed male C57BL/6 J mice were fasted for 6hrs and subjected to a GTT^47^. Four or 36 hrs before the injection of glucose (2 g/kg body weight), mice received an ip. or iv. injection of freshly prepared AdEVs or vehicle. Blood glucose levels were repeatedly measured from the tail vein using FreeStyle FREEDOM Lite glucometers. Plasma insulin was analyzed by ELISA (Crystal Chem Inc.).

### Statistical analyses

Statistical analyses were performed using GraphPad Prism8. Data are shown as mean ± SEM. Differences between groups were either assessed by unpaired t-tests (2 groups), 1-way ANOVA or 2-way ANOVA (multiple groups) with time and treatment as co-variates followed by post-hoc multiple comparisons testing. Details are given in the figure legends. A *P*-value <0.05 was considered statistically significant.

### Reporting summary

Further information on research design is available in the Nature Portfolio Reporting Summary linked to this article.

## Supplementary information

Supplementary Information

Reporting Summary

## Data Availability

The mass spectrometry proteomics data has been deposited to the ProteomeXchange Consortium via the PRIDE partner repository^48^ (https://www.ebi.ac.uk/pride/) with the dataset identifier PXD037809. All other data generated or analyzed during this study are included in this published article (and its supplementary information files). Source data are provided with this paper.

## Source data

Source Data

## References

[1] Thery C (2018). Minimal information for studies of extracellular vesicles 2018 (MISEV2018): a position statement of the International Society for Extracellular Vesicles and update of the MISEV2014 guidelines. J. Extracell. Vesicles.

[2] Todkar K (2021). Selective packaging of mitochondrial proteins into extracellular vesicles prevents the release of mitochondrial DAMPs. Nat. Commun..

[3] D’Acunzo P (2021). Mitovesicles are a novel population of extracellular vesicles of mitochondrial origin altered in Down syndrome. Sci. Adv..

[4] van Niel G, D’Angelo G, Raposo G (2018). Shedding light on the cell biology of extracellular vesicles. Nat. Rev. Mol. Cell Biol..

[5] Huang Z, Xu A (2021). Adipose extracellular vesicles in intercellular and inter-organ crosstalk in metabolic health and diseases. Front Immunol..

[6] Hartwig S (2019). Exosomal proteins constitute an essential part of the human adipose tissue secretome. Biochim Biophys. Acta Proteins Proteom..

[7] Ying W (2017). Adipose tissue macrophage-derived exosomal miRNAs can modulate in vivo and in vitro insulin sensitivity. Cell.

[8] Yu Y (2018). Adipocyte-derived exosomal MiR-27a induces insulin resistance in skeletal muscle through repression of PPARgamma. Theranostics.

[9] Zhang Y (2016). Adipocyte-derived microvesicles from obese mice induce M1 macrophage phenotype through secreted miR-155. J. Mol. Cell Biol..

[10] Pan Y (2019). Adipocyte-secreted exosomal microRNA-34a inhibits M2 macrophage polarization to promote obesity-induced adipose inflammation. J. Clin. Invest..

[11] Gao H, Luo Z, Jin Z, Ji Y, Ying W (2021). Adipose tissue macrophages modulate obesity-associated beta cell adaptations through secreted miRNA-containing extracellular vesicles. Cells.

[12] Gesmundo I (2021). Adipocyte-derived extracellular vesicles regulate survival and function of pancreatic beta cells. JCI Insight.

[13] Surwit RS, Kuhn CM, Cochrane C, McCubbin JA, Feinglos MN (1988). Diet-induced type II diabetes in C57BL/6J mice. Diabetes.

[14] Kleinert M (2018). Animal models of obesity and diabetes mellitus. Nat. Rev. Endocrinol..

[15] Kowal J (2016). Proteomic comparison defines novel markers to characterize heterogeneous populations of extracellular vesicle subtypes. Proc. Natl Acad. Sci. USA.

[16] Kahn CR, Wang G, Lee KY (2019). Altered adipose tissue and adipocyte function in the pathogenesis of metabolic syndrome. J. Clin. Invest.

[17] Friedman JM (2019). Leptin and the endocrine control of energy balance. Nat. Metab..

[18] Yang Q (2005). Serum retinol binding protein 4 contributes to insulin resistance in obesity and type 2 diabetes. Nature.

[19] Shoelson SE, Lee J, Goldfine AB (2006). Inflammation and insulin resistance. J. Clin. Invest.

[20] Deng T (2013). Class II major histocompatibility complex plays an essential role in obesity-induced adipose inflammation. Cell Metab..

[21] Divanovic S (2005). Negative regulation of Toll-like receptor 4 signaling by the Toll-like receptor homolog RP105. Nat. Immunol..

[22] Yamauchi T (2007). Targeted disruption of AdipoR1 and AdipoR2 causes abrogation of adiponectin binding and metabolic actions. Nat. Med..

[23] Ussar S, Bezy O, Bluher M, Kahn CR (2012). Glypican-4 enhances insulin signaling via interaction with the insulin receptor and serves as a novel adipokine. Diabetes.

[24] Beaton N (2015). TUSC5 regulates insulin-mediated adipose tissue glucose uptake by modulation of GLUT4 recycling. Mol. Metab..

[25] Czech MP, Tencerova M, Pedersen DJ, Aouadi M (2013). Insulin signalling mechanisms for triacylglycerol storage. Diabetologia.

[26] Fink LN (2013). Expression of anti-inflammatory macrophage genes within skeletal muscle correlates with insulin sensitivity in human obesity and type 2 diabetes. Diabetologia.

[27] Ong SE (2002). Stable isotope labeling by amino acids in cell culture, SILAC, as a simple and accurate approach to expression proteomics. Mol. Cell Proteom..

[28] Campbell JE, Newgard CB (2021). Mechanisms controlling pancreatic islet cell function in insulin secretion. Nat. Rev. Mol. Cell Biol..

[29] Prentki M, Matschinsky FM, Madiraju SR (2013). Metabolic signaling in fuel-induced insulin secretion. Cell Metab..

[30] Sassmann A (2010). The Gq/G11-mediated signaling pathway is critical for autocrine potentiation of insulin secretion in mice. J. Clin. Invest.

[31] Wan QF (2004). Protein kinase activation increases insulin secretion by sensitizing the secretory machinery to Ca2+. J. Gen. Physiol..

[32] Sacco F (2016). Glucose-regulated and drug-perturbed phosphoproteome reveals molecular mechanisms controlling insulin secretion. Nat. Commun..

[33] Ruan J (2012). DiR-labeled embryonic stem cells for targeted imaging of in vivo gastric cancer cells. Theranostics.

[34] Flaherty SE, Grijalva A, Xu X, Ables E, Nomani A, Ferrante AW (2019). A lipase-independent pathway of lipid release and immune modulation by adipocytes. Science.

[35] Essandoh K (2015). Blockade of exosome generation with GW4869 dampens the sepsis-induced inflammation and cardiac dysfunction. Biochim. Biophys. Acta.

[36] Nolan CJ, Damm P, Prentki M (2011). Type 2 diabetes across generations: from pathophysiology to prevention and management. Lancet.

[37] Deng ZB (2009). Adipose tissue exosome-like vesicles mediate activation of macrophage-induced insulin resistance. Diabetes.

[38] Wiklander OP (2015). Extracellular vesicle in vivo biodistribution is determined by cell source, route of administration and targeting. J. Extracell. Vesicles.

[39] Crewe C, Scherer PE (2022). Intercellular and interorgan crosstalk through adipocyte extracellular vesicles. Rev. Endocr. Metab. Disord..

[40] Montgomery MK (2019). Mitochondrial dysfunction and diabetes: is mitochondrial transfer a friend or foe?. Biol. (Basel).

[41] Clement E (2020). Adipocyte extracellular vesicles carry enzymes and fatty acids that stimulate mitochondrial metabolism and remodeling in tumor cells. EMBO J..

[42] Thomou T (2017). Adipose-derived circulating miRNAs regulate gene expression in other tissues. Nature.

[43] Kulak NA, Pichler G, Paron I, Nagaraj N, Mann M (2014). Minimal, encapsulated proteomic-sample processing applied to copy-number estimation in eukaryotic cells. Nat. Methods.

[44] Humphrey SJ, Karayel O, James DE, Mann M (2018). High-throughput and high-sensitivity phosphoproteomics with the EasyPhos platform. Nat. Protoc..

[45] Grosche A (2016). The proteome of native adult muller glial cells from murine retina. Mol. Cell Proteom..

[46] Volta F (2019). Glucose homeostasis is regulated by pancreatic beta-cell cilia via endosomal EphA-processing. Nat. Commun..

[47] Stemmer K (2015). FGF21 is not required for glucose homeostasis, ketosis or tumour suppression associated with ketogenic diets in mice. Diabetologia.

[48] Perez-Riverol Y (2022). The PRIDE database resources in 2022: a hub for mass spectrometry-based proteomics evidences. Nucleic Acids Res..

