# Supplementary material for: Adipocyte-derived extracellular vesicles increase insulin secretion through transport of insulinotropic protein cargo
Source: Nat Commun. 2023 Feb 9;14:709. doi: 10.1038/s41467-023-36148-1 (PMC9911726; doi:10.1038/s41467-023-36148-1)
Supplement: Supplementary file 1 — Supplementary Information [file 41467_2023_36148_MOESM1_ESM.pdf]

SUPPLEMENTARY MATERIAL

Adipocyte-derived extracellular vesicles increase insulin secretion through transport of insulinotropic protein cargo

Konxhe Kulaj[1,2,3 #], Alexandra Harger[1,2 #], Michaela Bauer[1,2,3 #], Özüm S. Caliskan[1,2 #], Tilak Kumar Gupta[4], Dapi Menglin Chiang[5], Edward Milbank[1,3], Josefine Reber[6], Angelos Karlas[6,7,8,9], Petra Kotzbeck[1,10], David N. Sailer[1,11], Francesco Volta[12,13], Dominik Lutter[1,2], Sneha Prakash[1,2], Juliane Merl-Pham[14], Vasilis Ntziachristos[6,7,9], Hans Hauner[15,16], Michael W. Pfaffl[5], Matthias H. Tschöp[1,2,17], Timo D. Müller[1,2], Stefanie M. Hauck[14], Benjamin D. Engel[18,19], Jantje M. Gerdes[12], Paul T. Pfluger[1,2,20,21], Natalie Krahmer[1,2], Kerstin Stemmer[1,2,3 *]

[1] Institute for Diabetes and Obesity, Helmholtz Zentrum München, Neuherberg, Germany
[2] German Center for Diabetes Research (DZD), Neuherberg, Germany
[3] Molecular Cell Biology, Institute for Theoretical Medicine, University of Augsburg, Augsburg, Germany
[4] Department of Molecular Structural Biology, Max Planck Institute of Biochemistry, Martinsried, Germany
[5] Division of Animal Physiology and Immunology, School of Life Sciences Weihenstephan, Technical University of Munich, Munich, Germany
[6] Institute of Biological and Medical Imaging, Helmholtz Zentrum München, Neuherberg, Germany
[7] Chair of Biological Imaging at the Central Institute for Translational Cancer Research (TranslaTUM), School of Medicine, Technical University of Munich, Munich, Germany
[8] Department for Vascular and Endovascular Surgery, Klinikum rechts der Isar, Technical University of Munich, Munich, Germany
[9] DZHK (German Centre for Cardiovascular Research), Partner Site Munich Heart Alliance, Munich, Germany
[10] Department of Surgery, Division of Plastic, Aesthetic and Reconstructive Surgery, Medical University of Graz, Graz, Austria
[11] Institute of Molecular Oncology and Functional Genomics, School of Medicine, Technical University of Munich, Munich, Germany
[12] Institute of Diabetes and Regeneration Research, Helmholtz Zentrum München, Neuherberg, Germany
[13] Department of Medicine and Surgery, University of Parma, Parma, Italy
[14] Metabolomics and Proteomics Core, Helmholtz Zentrum München, Neuherberg, Germany
[15] Institute for Nutritional Medicine, School of Medicine, Technical University of Munich, Munich, Germany
[16] ZIEL - Institute for Food & Health, Technical University of Munich, Freising, Germany
[17] Division of Metabolic Diseases, Department of Medicine, Technical University of Munich, Munich, Germany
[18] Helmholtz Pioneer Campus, Helmholtz Zentrum München, Neuherberg, Germany
[19] Biozentrum, University of Basel, Basel, Switzerland
[20] Research Unit NeuroBiology of Diabetes, Helmholtz Zentrum München, Neuherberg, Germany
[21] Chair of Neurobiology of Diabetes, TUM School of Medicine, Technical University of Munich, Munich, Germany

[*] Corresponding author
[#] Equal contribution

Corresponding address:
Kerstin Stemmer, Molecular Cell Biology, Institute for Theoretical Medicine, University of Augsburg, Universitätsstraße 2, 86159 Augsburg, Germany
Email: kerstin.stemmer@med.uni-augsburg.de; Phone: +49- 821-598-71116

SUPPLEMENTARY MATERIAL AND METHODS

AdEV isolation from murine eWAT by dUC and SEC

Per biological replicate, up to 10 fat pads (ca. 4 g) were coarsely minced in pre-warmed DMEM GlutaMAX (Thermo Fisher Scientific), incubated in 0.075% collagenase and 1% BSA for 40 minutes and passed through a 100 µm mesh. Adipocytes and the stromal-vascular fraction (SVF) were separated by centrifugation at 100 g for 10 minutes. The adipocyte layer was incubated in medium (DMEM GlutaMAX, with 0.2% BSA (low endotoxin)) at 37°C in a humidified atmosphere for 2 hours. Conditioned media were centrifuged for 10 minutes at 1000 g followed by centrifugation at 10.000 g for 30 minutes to remove debris. Supernatants were subjected to differential ultracentrifugation (dUC) or size exclusion chromatography (SEC) to isolate AdEVs. For dUC isolation, supernatants from eWAT explant cultures were centrifuged at 100,000 g for 70 minutes, pellets were washed in phosphate-buffered saline (PBS) and re-centrifuged at 100,000 g for 70 minutes in an Optima™ MAX centrifuge using a TLA-55 rotor (adjusted k-factor of 125, acceleration and brake at maximum) and 1.5 mL Microfuge® polyallomer tubes (Beckman Coulter). EV-enriched fractions were re-suspended in PBS for molecular analyses. For SEC isolation, supernatants were concentrated via Pierce 20 mL 100kDa cutoff ultrafiltration units and centrifugation at 3.000x g for 20 minutes at room temperature. Samples were collected in 500 µl PBS, subjected to IZON qEV70 original size exclusion columns (IZON) and eluted with particle-free PBS. Fractions 7-10 were collected via an IZON Automatic Fraction Collector and concentrated using Amicon 2 mL 10 kDa cutoff ultrafiltration units.

Human adipose tissue processing for AdEV and SVF-EV isolation

Human adipose tissue samples were obtained from female patients undergoing liposuction. After the surgical procedures, tissue samples were transferred into sterile vessels containing medium (DMEM/F12, GlutaMAXTM, Thermo Fisher Scientific GmbH, Dreieich, Germany) and kept at 4°C. To remove blood contaminants and medium residues the tissue was washed with PBS, followed by 25 minutes of incubation in 0.075% Collagenase IV and 1% BSA (low endotoxin) at 37°C. Following digestion and filtration through a 100 µm mesh, the human adipocytes were separated from the SVF by centrifugation at 100 g for 10 minutes and isolated adipocytes were incubated in medium (DMEM GlutaMAX, with 0.2% BSA (low endotoxin)) at 37°C in a humidified atmosphere for 2 hours. For isolation of EVs originating from the SVF, the cell pellet was first incubated with erythrocyte lysis buffer (154 mM NH_4Cl, 10 mM $KHCO_3$, 0.1 mM EDTA) to lyse contaminating red blood cells before the incubation in medium for 2 hours. The Ad- or SVF-EVs secreted into the medium were isolated using differential centrifugation as already described for murine adipose tissue. EVs were further purified by size exclusion chromatography using Exo-spin columns (Cell Guidance Systems) according to the manufacturer's protocol.

Isolation of murine pancreatic islets

Islets of Langerhans were isolated from 3-month-old male C57BL/6J mice by collagenase digestion. Briefly, after killing the mice by cervical dislocation the pancreas was perfused with 1 mg/ml Collagenase P, dissolved in G-solution (HBSS with 25 mM HEPES, pH 7.4, supplemented with 0.5% BSA) via injection into the common bile duct. To ensure a correct perfusion of the entire pancreas, the duodenum was clamped at the ampulla of Vater with a serrefine. The perfused

pancreas was carefully removed and digested in a water bath at 37°C for 14 min. Islets were hand-picked multiple times to ensure a high purity and were maintained overnight (ON) in suspension dishes with culture medium (RPMI 1640, 10% FBS (not inactivated), 100 U/ml penicillin, 100 µg/ml streptomycin) to recover from the isolation procedure.

Nanoparticle tracing analysis (NTA)

A ZetaView® Nanoparticle Tracking Analyzer PMX 110 (Particle Metrix GmbH) was set at two cycles per measurement, scanning 11 positions each and capturing 60 frames per second. Pre-acquisition parameters were: sensitivity 80, shutter 70, cell temperature 25°C, trace length 15. Videos were analyzed by the inbuilt ZetaView Software 8.05.11 SP1 using minimum particle brightness 20, minimum size 10 pixels, maximum size 1000 pixels, PSD nm/class 10 and PSD classes/decade 10.

Transmission electron cryo- microscopy (Cryo-TEM)

Fresh EV samples (4 µl) were applied to glow discharged, holey carbon-coated copper grids (R 2/1, 200 mesh, Quantifoil Micro Tools) and plunge-frozen in a liquid ethane/propane mixture using a Vitrobot Mark 4 (FEI, Thermo Fisher Scientific). Blotting chamber conditions were set to 4°C and 95% humidity, and grids were blotted using blot force 8 and a blot time of 6 seconds. Grids were stored in liquid nitrogen until usage. Images were recorded with SerialEM software [1] on a Tecnai G2 Polara transmission electron microscope (FEI, Thermo Fisher Scientific) equipped with a field emission gun operated at 300 kV, a post-column energy filter (Gatan), and a 3838 × 3710 Gatan K2 Summit direct detection camera operated in counting mode. For each image, 20 frames were acquired over 10 seconds, with a total dose of ~45 e-/Å^2 and a calibrated pixel size of 2.36 Å. A total of 697 images were

recorded, using a defocus range of −2 to −5 µm. The frame stacks were subjected to alignment with 5x5 patches and dose filtering using MotionCor2 [2]. Images were binned to a pixel size of 7.04 Å for display and measurement [3].

Western blot analysis

Equal amounts of (i) SEC isolated vesicles (2.3 x 10^9 particles), of (ii) dUC EVs (30 μg) and of (iii) SVF/adipocyte fractions (10 μg) were diluted in RIPA buffer supplemented with protease inhibitors (Roche) and 4x loading buffer was added. The samples were denatured at 95 °C, 350 rpm, for 10 minutes. The proteins were separated via 12% (EV samples) or 4-20% (for SVF/adipocyte samples) precast gels (Criterion™ TGX precast gels, Bio-Rad Laboratories) and Tris-Glycine/SDS electrophoresis buffer (Serva Electrophoresis) at 70 V. Proteins were transferred from the gel to a Roti-polyvinylidene difluoride (PVDF) membrane (Roth) using a gel sandwich and a wet transfer protocol for 2:30 hours at 70 V at 4 °C. Briefly, transfer cassettes, sponge, whatman paper, gel and the membrane were arranged into a Criterion™ Blotter according to the manufacturer's instructions (Bio-Rad Laboratories, Inc.). Membranes were then blocked, washed in Roti-TBS-T and later incubated overnight at 4°C and under shaking with the primary antibodies against TSG-101 (Sigma Aldrich, 1/1000, HPA006161), RPL-5 (Cell Signaling, 1/1000, 14568), CD63 (Santa Cruz, 1/1000, sc-5275, clone MX-49.129.5), F4/80 (Cell Signaling, 1/1000, 70076T, clone D2S9R) and GAPDH (Millipore, 1/3000, CB1001, clone 6C5) and the appropriate secondary antibody IgG-HRP (Santa Cruz, 1/10000, goat anti-mouse: sc-2005 or goat anti-rabbit: sc-2030) for 60 minutes at RT while shaking. Membranes were developed using ECL solution (Clarity Max Western ECL Substrate, Bio-Rad Laboratories, Inc.) and the ChemiDoc Imaging System (Bio-Rad

Laboratories, Inc.). Quantification of total protein concentrations in each lane for confirming equal sample loading was determined using Coomassie Brilliant Blue R 250 (Serva Electrophoresis) for 4 minutes. Membranes were washed with destain solution (50% methanol, 10% acetic acid and 40% distilled water) for 10 minutes then with water several times and air-dried.

MIN6 mouse clonal β-cells were provided by Prof. J. Miyazaki (Osaka University, Japan) and grown in Dulbecco's modified eagle's medium (DMEM) with glutaMAX containing 25 mM glucose (Thermo Fisher Scientific, Waltham, MA, USA) supplemented with 15% heat-inactivated HycloneTM Fetal Bovine Serum (Thermo Fisher Scientific, Waltham, MA, USA), 72 µM 2-mercaptoethanol, 100 µg/mL penicillin, and 100 µg/mL streptomycin. For cellular proteome and paralleled secretome analyses, 4* 10^4 MIN6 cells were seeded into 96 well plates. After 4 days, each well was treated with either vehicle, or 2.3*10^9 AdEV particles from lean or obese mice dissolved in FBS-free SILAC containing medium for 6 hours. Supernatants were then collected for heavy amino acid labeled secretome analyses.

Sample preparation for proteome and secretome analysis

Adhesive MIN6 cells were washed 2x in ice-cold TBS, harvested and prepared for liquid chromatography-mass spectrometry (LC-MS/MS) as described previously [4]. Briefly, Min6 cells and supernatant samples were lysed in SDC lysis buffer (2% SDC, 100 mM Tris-HCl pH 8.5) at 95°C for 10 minutes at 1.000 rpm, sonicated in high mode (30 seconds OFF, 30 seconds ON) for 10 cycles (Bioruptor® Plus; Diagenode). The protein concentration was determined by the bicinchoninic acid (BCA) assay and 25 µg of protein were used for further analysis. Samples were treated with TCEP and CAA (final concentrations of 10 mM and 40 mM, respectively) at 45°C for 10 minutes with 1000-rpm shake, in dark, and digested with trypsin and

LysC (1:40, protease:protein ratio) overnight at 37°C, 1,000 rpm shake. Later, peptides were acidified with 2% TFA, isopropanol with 1:1 volume-to-volume ratio. Custom-made StageTips with three layers of styrene divinylbenzene reversed-phase sulfonate (SDB-RPS; 3 M Empore) membranes were used for desalting and purification of the acidified samples. Peptides were loaded on the activated (100% ACN, 1% TFA in 30% Methanol, 0.2% TFA, respectively) StageTips, run through the SDB-RPS membranes, and washed by EtOAc including 1% TFA, isopropanol including 1% TFA, and 0.2% TFA, respectively. Peptides eluted from the membranes with 60 µl elution buffer (80% ACN, 1.25% NH_4OH) were dried by vacuum centrifuge (40 minutes at 45°C) and reconstituted in 6 µl of loading buffer (2% ACN, 0.1% TFA). Peptide concentration was estimated via optical measurement at 280 nm (Nanodrop 2000; Thermo Scientific) and 500 ng material were loaded onto 50 cm in-house packed HPLC column (75 µm inner diameter, packed with 1.9 µm with C18 Reprosil particles (Dr. Maisch GmbH) at 60°C) for LC-MS/MS analysis.

Sample preparation for phosphoproteomics

For phospho-proteome analyses, 5* 10^5 MIN6 cells were seeded in 6-well plates. Each well was treated with PBS (vehicle control) or 2.3* 10^{10} AdEVs from DIO mice for 6 hours in FBS-free high glucose DMEM supplemented with penicillin/streptomycin and β-mercaptoethanol. Subsequently, cells were transferred for 105 minutes to 2 mM low glucose Krebs-Ringer buffer (KRB). Cells were then switched to high glucose (25 mM) KRB for 15 minutes washed (2x with ice cold TBS) and harvested for LC-MS/MS analyses.

Samples for phosphoproteomics analyses were prepared by the EasyPhos workflow [5] with minor changes. Briefly, samples were lysed in 4% SDC, 100 mM Tris-HCl pH 8.5 buffer at 95°C for 10 minutes. Samples were sonicated, reduced, alkylated

and digested as described for the total proteome sample preparation. Digested samples were mixed with ACN and enrichment buffer (36% TFA, 3 mM KH_2PO_4), respectively, for 30 seconds with 2,000 rpm shake. Samples were cleared for 15 minutes at 20,000 g centrifuge, and supernatants were transferred into new tubes. Enrichment of the phophopeptides were done with TiO_2 beads. TiO_2 beads were re-suspended in loading buffer (80% ACN, 6% TFA), added into samples (1:12, protein:bead ratio) and incubated at 40°C, for 5 minutes, at 2,000 rpm. Beads were pelleted by 1 minute centrifugation at 2,000 g, supernatant was discarded and pellets were washed for 5 times with wash buffer (60% ACN, 1% TFA). Washed beads were re-suspended in transfer buffer (80% ACN, 0.5% acetic acid), loaded on top of double layer C8 StageTips, and centrifuged to dryness (5 minutes, 1,000xg). Phosphopeptides were eluted from the C8 (C8; 3 M Empore) membranes into PCR tubes with 60 µl of elution buffer (40% ACN, 3.75% NH_4OH) and concentrated in a SpeedVac for 15 minutes at 45 °C. Concentrated samples were acidified with 1% TFA and purified by SDB-RPS StageTips. In the final step, phosphopeptides were reconstituted in 6 µl of loading buffer (2% ACN, 0.1% TFA) and without measuring peptide concentration, 5 µl of the sample was loaded into the column for LC-MS/MS analyses.

LC-MS/MS analysis of the proteome and secretome

For human proteome studies, 0.5 µg peptides of human EV samples were analyzed using a Q-Exactive HF mass spectrometer coupled to an Ultimate 3000 nano-HPLC (Thermo Scientific) as previously reported [6]. For all other proteomes, 0.5 µg of peptides were analyzed using the EASY-nLC 1200 (Thermo Fisher Scientific) connected to Orbitrap Exploris 480 Mass Spectrometer (Thermo Fisher Scientific) and a nano-electrospray ion source (Thermo Fisher Scientific). FAIMS Pro™ with

Orbitrap Fusion ™ module was used in analysis of proteome and secretome samples with two different compensation voltages (CV) (-50V and -70V).
Peptides from cellular lysates or from supernatant samples were loaded at 60°C onto a 50 cm, 75 μm inner diameter, in-house packed HPLC column with 1.9 μm with C18 Reprosil particles (Dr. Maisch GmbH) and separated by reversed-phase chromatography using a binary buffer system (0.1% formic acid (buffer A) and 80% ACN in 0.1% formic acid (buffer B) over a 120 minutes gradient (5-30% buffer B over 95 min, 30-65% buffer B over 5 minutes, 65-95% buffer B over 5 minutes and wash with 95% buffer B for 5 minutes) at a flow rate of 300 nl/min. MS data were acquired using a one-second cycle time data-dependent MS/MS scan method. Full scan MS targets were in a 300–1650 m/z scan range with $3*10^6$ charges and data was acquired in 60,000 at m/z 200 resolution with 25 ms maximum injection time. Precursor ions for MS/MS scans were fragmented by higher-energy C-trap dissociation (HCD) with a normalized collision energy of 30. MS/MS scan sets were 15,000 at m/z 200 resolution with an ion target value of $1*10^5$ and a maximum injection time of 28 ms. Dynamic exclusion was set to 120 seconds.

LC-MS/MS analysis of the phospho-proteome

Phosphoproteome samples were analyzed without FAIMS. Phosphopeptides of the first phospho-experiment (Fig 4 f and g) were eluted with a 120 minutes gradient (5–25% buffer B over 70 minutes, 25–50% buffer B over 30 minutes, 50-95% buffer B over 5 minutes, 5 minutes wash with 95% buffer B, 95-5% buffer B over 5 minutes and 5 minutes wash with 5% buffer B) with 20 ms maximum injection time. Phosphopeptides of the second phospho-experiment were eluted with a 140 minutes gradient (5–25% buffer B over 85 minutes, 25–50% buffer B over 35 minutes, 50-95% buffer B over 5 minutes, 5 minutes wash with 95% buffer B, 95-5% buffer B over

5 minutes and 5 minutes wash with 5% buffer B) with 20 ms maximum injection time. Full MS scans were acquired in 300–1650Th range with 3*10^{6} charges at 60,000 resolution with 100 ms and 80 ms maximum injection time for the first and the second phospho-experiments, respectively. Precursor ions for MS/MS scans were fragmented by higher-energy C-trap dissociation (HCD) with a normalized collision energy of 27. MS/MS scans were acquired 15,000 at m/z 200 resolution with an ion target value of 1*10^{5}, a maximum injection time of 50 ms. Dynamic exclusion was set to 40 seconds for both phosphor-experiments.

Proteome and secretome data processing and analysis

The raw data were processed with MaxQuant version 1.6.14.0. Default settings were kept if not stated otherwise. FDR 0.01 was used for filtering at protein, peptide and modification level. As variable modifications, acetylation (protein N-term) and oxidized methionine (M), as fixed modifications, carbamidomethyl (C) were selected. Trypsin/P and LysC proteolytic cleavages were added. Maximum missed cleavages allowed for protein analysis was two. ‘‘Match between runs’’ and Label free quantitation (LFQ) were enabled and all searches were performed against the mouse or human Uniprot FASTA database (2019). Perseus (version 1.6.14.0) was used for bioinformatics analysis. UniProtKB, Gene Ontology (GO), and the Kyoto Encyclopedia of Genes and Genomes (KEGG) annotations were included in the analysis. Data were visualized using Adobe Illustrator or Graphpad Prism (version 8.0).

In Perseus, LFQ intensities were used for proteome and secretome analyses. The raw data were filtered for “only identified by site”, “reverse”, and “potential contaminant”. Protein intensities were log2 transformed for further analyses. Quantified proteins were filtered either for at least two or 70% valid values among

biological replicates in at least one condition. Quantified proteins for generating the correlation profile of AdEVs and eWAT lysates (n=5, figure 2f) were filtered with a minimum of 3 valid values in each group. Groups were averaged with a minimum of two valid values in at least one group in case of Venn diagram generation. Missing values were imputed (Gaussian normal distribution with a width of 0.3 and a downshift of 1.8) in order to obtain PCA, hierarchical clusters, volcano plots and to apply the t-test. The default imputation method in Perseus assumes that missing values represent low abundance measurements [7, 8]. Random values are drawn from a distribution meant to simulate expression below the detection limit. The parameters of this distribution are optimized to simulate a typical abundance region that the missing values would have if they had been measured. The Perseus default imputation approach is column-wise imputation with each column representing the different samples, which we applied to each expression sample separately. This strategy is recommended when the compared proteomes (EV and cell proteomes) might be different from each other ("https://github.com/JurgenCox/perseus-plugins/blob/master/PerseusPluginLib/Impute/ReplaceMissingFromGaussian.cs").

Two-sample test with student t-test statistics (permutation-based FDR = 0.1; s0 = 0) was performed in Perseus and significant proteins were hierarchical clustered by applying Euclidean as a distance measure for row clustering after normalization of median protein abundances of biological replicates by z-score. Fisher's Exact GOBP, GOCC, GOMF and KEGG Term enrichment of significant proteins was performed against mouse database (gene list with 21846 entries) in Perseus (Benjamini Hochberg FDR truncation with 0.05 threshold value).

For background subtraction to correct for possible unlabeled host cell proteins, "vehicle" group is used as "background". Median of the raw data of Vehicle group replicates was taken with condition of minimum two valid values per group. The

median value was subtracted from each replicate of other groups. The subtracted values were processed as explained above and used for the downstream analysis. Exclusive and shared proteins among the groups (Venn diagram analysis in Figure 3 and 4) were determined by taking the median of the replicates (minimum two valid values per group) for the filtered and log2 transformed raw (non-imputed) data. Once the median values were obtained, “Venn Diagram” option in Perseus was applied. Samples named BMI9309A3MA4-40-325267, BMI9309A2MA3-40-325260, and BMI9104A10MA4-40-325147 in human raw data were removed while doing statistical analysis on Perseus because they either belong to a different gender or not belong to AT or SVF groups.

Phosphoproteome data processing and analysis

Data processing for phosphoproteome analysis follows the proteome analysis protocol with some modification. Briefly, phospho (STY) was included in analysis of phosphosites additionally to the mentioned variable modifications in the proteome analysis section. Maximum missed cleavages were set as four for the phosphorylation analyses. In Perseus, intensities were used for phosphoproteome analysis. The raw data were filtered for “reverse”, and “potential contaminant”. Expand site table and width adjustment were applied in phosphoproteome analysis. Matrixes corresponding to “light” and “heavy” phopshoproteins were matched with outer option.

MicroRNA isolation and profiling and AdEVs loading with miRNA

MicroRNA isolation from AdEVs and profiling on Exiqon rodent miRNA PCR panel was conducted as fee for service at Exiqon A/S, Denmark. Briefly, isolated AdEVs (3 – 7 µg) were lysed in Qiazol® lysis reagent (Qiagen) and pre-purified by phenol

(Qiazol)/chloroform extraction. Nucleic acids in the aqueous phase were precipitated with 100% ethanol and total RNA was extracted using the Qiagen miRNeasy® Mini Kit (Qiagen) according to standard protocols. RNA was reverse transcribed in 50 µL reactions using the miRCURY LNA™

Universal RT microRNA PCR Polyadenylation and cDNA synthesis kit (Exiqon). cDNA was diluted 50-fold and assayed in 10 µL PCR reactions according to the protocol for miRCURY LNA™ Universal RT microRNA PCR; each microRNA was assayed once by qPCR on the microRNA Ready-to-Use PCR Mouse&Rat panel I (Exiqon rodent miRNA PCR panel) using ExiLENT SYBR® Green master mix. Negative controls excluding template from the reverse transcription reaction were profiled like the samples. The amplification was performed in a LightCycler® 480 Real-Time PCR System (Roche) in 384 well plates. The amplification curves were analyzed using the Roche LC software, both for determination of Cq (by the 2nd derivative method) and for melting curve analysis. The amplification efficiency was calculated using algorithms similar to the LinReg software. All assays were inspected for distinct melting curves and the Tm was checked to be within known specifications for the assay. Furthermore, assays must be detected with 5 Cqs less than the negative control, and with Cq < 37 to be included in the data analysis. Data that did not pass these criteria were omitted from any further analysis. Cq was calculated as the 2nd derivative. Using NormFinder the best normalizer was found to be the average of assays detected in all samples. All data was normalized to the average of assays detected in all samples (average –assay Cq).

For the AdEV loading with miRNAs, freshly isolated DIO AdEVs (150 µg) were transfected with 20 pmol miRNA Negative Control #1 (Assay ID: 4464058) or a miRNA mixture of miR-29a-3p (Assay ID:MC12499), miR-200a-3p (Assay ID:MC10991), miR-218-5p (Assay ID:MC10328) and miR-322-5p (Assay

ID:MC11080); (all mirVana™ miRNA Mimics, Thermo Fisher Scientific) using the Exo-Fect™ Exosome Transfection Kit. Transfected EVs were resuspended in 150 µl PBS and incubated for 6 hours with 150 murine islets isolated the prior day followed by an in vitro dynamic insulin release assay.

Uptake of small RNAs into murine pancreatic islets

500 µl of plain RPMI 1640 medium were added with 15 µl of Texas Red end-labeled siRNA (System Biosciences) into two TC 35 suspension dishes (Sarstedt). Afterwards, 50 freshly isolated pancreatic islets were picked into each of the dishes with 2.5 mL growth medium (RPMI 1640, 10% FBS, 100 U/ml penicillin, 100 µg/mL streptomycin). The islets were imaged on a BZ-9000 microscope (Keyence) at different time-points, starting 6 hours post treatment and ending with an overnight exposure.

Cellular glucose uptake

For glucose uptake experiments, MIN6 cells transiently transfected with Green Glifon600 expression plasmid (Cat # 126207; Addgene) [9] were treated with $2.3*10^9$ DIO or lean AdEVs for 6 hours and subjected to baseline measurements every 44 seconds using a PHERAstar FS multi-mode microplate reader. Subsequently, cells were supplemented with either PBS without glucose (vehicle) or PBS plus 25 mM glucose, generating four different groups; MIN6 cells without glucose (vehicle), MIN6 cells with glucose (Vehicle+ Gluc), MIN6 cells with DIO AdEVs and glucose (DIO AdEVs + Gluc) and MIN6 cells with lean AdEVs and glucose (Lean AdEVs + Gluc). Glucose uptake rate was normalized to vehicle and well-specific baseline readings, and expressed as normalized fluorescence intensity.

Fluorescent AdEV uptake in vivo and in vitro

Freshly isolated EVs (20 µg in EV buffer) were incubated with the near-infrared fluorescent cyanine dye 1,1'-dioctadecyl-3,3,3',3'-tetramethylindotricarbocyanine iodide (DiR) diluted 1:500 in 1xPBS at 37°C, subsequently washed with 1x PBS and pelleted at 100.000x g for 130 minutes at 4°C. Stained EVs were re-suspended in EV buffer, quantified and immediately used for experiments. EV buffer subjected to the same labeling protocol served as a vehicle control. For in vivo tracking studies, 10 µg of DiR labeled AdEVs or DIR-vehicle control were diluted in PBS and injected intra peritoneally or intravenously into 12- to 16-week-old male C57BL/6J mice 4 and 24 hours prior to sacrifice followed by shaving and freezing of the mouse carcass at -20°C. After embedding in Tissue-Tek® O.C.T., mice were cryosliced along the long axis at steps of 100 µm and co-registered color and fluorescence cross-sectional images were captured at 800 nm in epi-illumination mode. For cryo-slicing and imaging we used a modified Leica cryotome (CM 1950, Leica Microsystems, Germany), equipped with a motorized multi-wavelength filter wheel and a CCD camera [10].

To study AdEV uptake into isolated murine pancreatic islets, 100 µg of feshly isolated DIO AdEVs were labeled with CellBrite™ Red Cytoplasmatic Membrane DiD Dye at 37°C for 30 minutes under rotation, washed with PBS and centrifuged at 100.000x g for 130 minutes at 4°C. Ten islets each were incubated with 10 µg of stained EVs or matching amounts of the DiD buffer for 6 hours before being fixed with 4% para-formaldehyde for 20 minutes at 37°C. Islets were permeabilized in 0.2% Triton-X in PBS (30 minutes) and blocked with 10% FBS and 3% donkey serum before an overnight incubation step at 4°C with primary anti-insulin antibody (1/300, Thermo Fisher Scientific, PA1-26938) diluted in blocking solution. Islets were subsequently washed with PBS and incubated for 2 hours with secondary antibody donkey anti-

guinea pig (1/300, Jackson ImmunoResearch Europe, UK706-545-148) diluted in blocking solution at RT. Islets were DAPI stained for 10 minutes and washed three times in PBS before Elvanol embedding. Pictures were taken with a confocal microscope (Leica TSC Sp5).

Metabolic phenotyping of GW4869 treated mice

Male C57BL/6J mice at an age of 5 weeks were given ad libitum access to HFD or chow diet for 24 weeks. Subsequently, mice were subjected to a combined indirect calorimetry system (TSE PhenoMaster, TSE Systems) that allows the simultaneous measurement of food intake, energy expenditure, respiratory exchange ratio, and locomotor activity. After 3 days of acclimatization to the air-tight cages, groups of lean and DIO mice (n=8) were ip. injected with 1 mg/kg GW4869, or vehicle (5% DMSO in 0.9% NaCl). Injections were performed once daily in the first hours of the light phase for 16 days. Fat and lean mass were analyzed at day 16 by nuclear magnetic resonance (EchoMRI). An ip. glucose tolerance test (ipGTT) was performed on day 13 after an initial fast of six hours. Glucose levels were measured before (0 mins) and 15, 30, 60 and 120 minutes after a 2.0 mg/kg ip. bolus of glucose. After 16 days of treatment mice were sacrificed by cervical dislocation. Epididymal fat pads were collected for EV isolation and quantification as described above.

SUPPLEMENTARY FIGURES

Supplementary Figure 1:

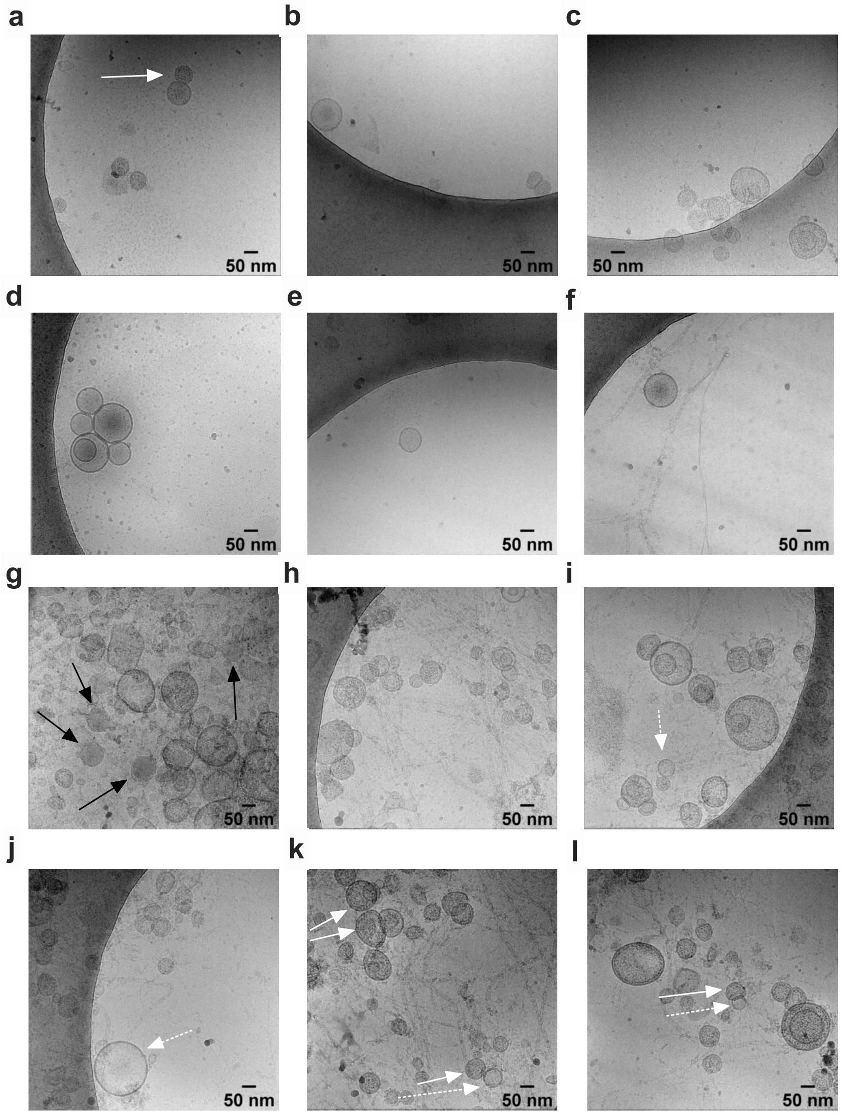

Transmission electron cryo-microscopy (cryo-TEM) images of EVs isolated from DIO and lean adipocytes via size exclusion chromatography (SEC) and differential ultracentrifugation (dUC)

A total of 697 cryo-TEM images were recorded with a Tecnai G2 Polara equipped with a Gatan K2 Summit detector. 954 SEC-derived AdEVs (**a-f**) and 4733 dUC-derived AdEVs (**g-l**) isolated from male diet-induced obese (DIO) C57BL/6J mice (two independent AdEVs isolations) were analyzed for their morphology. Only 60 (1,3%) monolayer vesicles were observed from dUC samples (**g**; black arrows), 98,7% of the dUC and 100% of the SEC AdEV population showed a clear presence of a lipid bilayer (**a-f**, **h-l**). Vesicles containing other vesicles were also visualized (**d**, **i**, **l**). Some vesicles were filled with electron-dense material in their lumen, whereas other vesicles had translucent lumens that appeared empty (**a**, **j**, **i**, **k**, **l**; white arrows and white dashed arrows, respectively). No distinct difference was identified in the morphology within DIO and lean AdEVs. Scale bars are 50 nm.

Supplementary Figure 2:

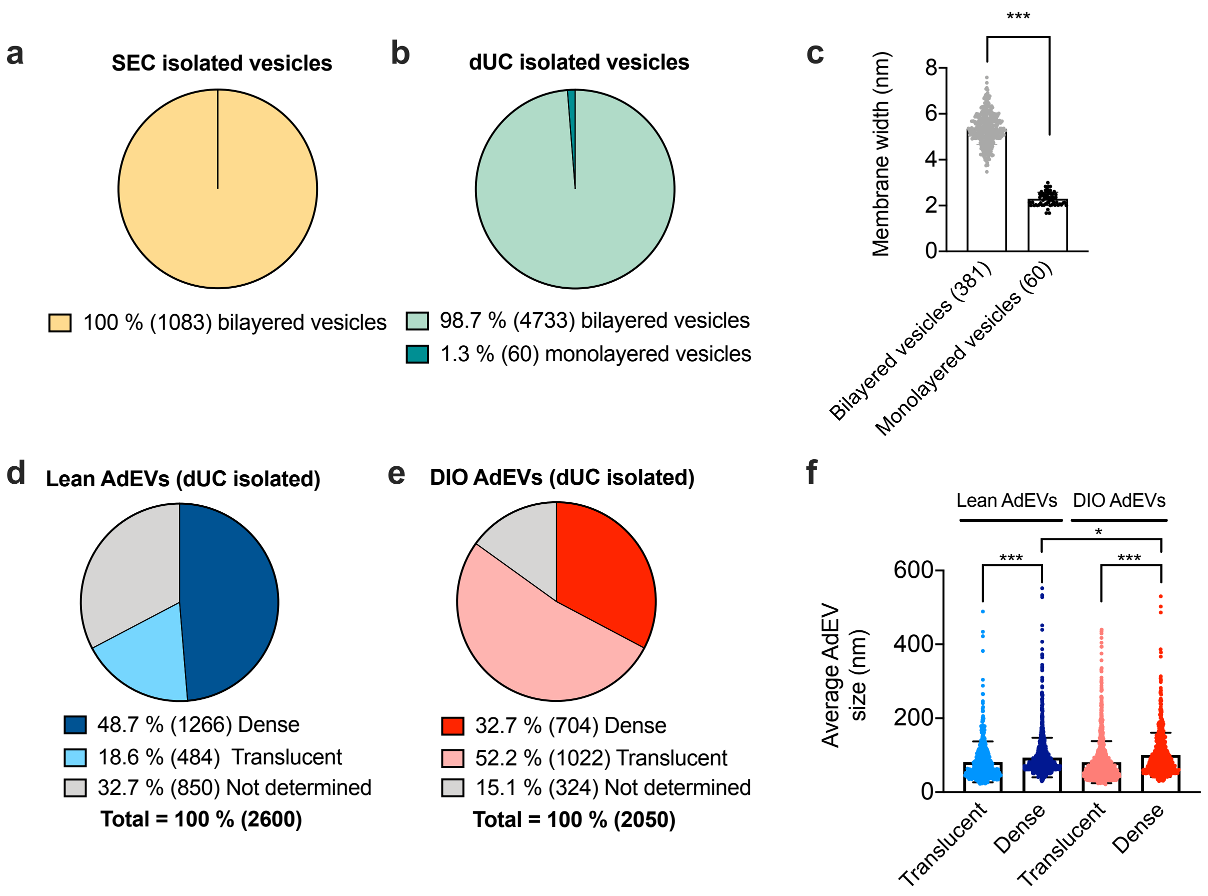

Structural analysis of AdEVs depicted by transmission electron cryo-microscopy (cryo-TEM)

(**a-b**) Quantification of total monolayered vs. bilayered vesicles from a total of 697 cryo-TEM images. Vesicles were isolated from the adipocyte fraction of diet-induced obese (DIO) C57BL/6J mice using (**a**) size exclusion chromatography (SEC) and (**b**) differential ultracentrifugation (dUC). (**c**) Membrane diameters of bilayered vs. monolayered vesicles. (**d-e**) Quantification of bilayer-encased AdEVs with dense or translucent lumina isolated with dUC from (**d**) lean mice or (**e**) DIO mice. Not determined vesicles were either cryo-fixed in too close proximity or placed on the grid, which prohibited further categorization. (**f**) Average AdEV sizes of bilayered translucent or dense AdEVs isolated with dUC from lean and DIO mice. Data are presented as mean values ± SEM (**c, f**). Significance was determined by unpaired two-tailed t-test (**c**) or one-way ANOVA and Sidak's multiple comparisons test (**f**). *p<0.05, ***p<0.001. Exact P values are (**c**) P<0.0001 and (**f**) P=0.0003 (Average lean AdEV size, translucent vs. dense), P<0.0001 (Average DIO AdEV size, translucent vs. dense), P=0.0219 (Average AdEV size, lean dense vs. DIO dense).

Supplementary Figure 3:

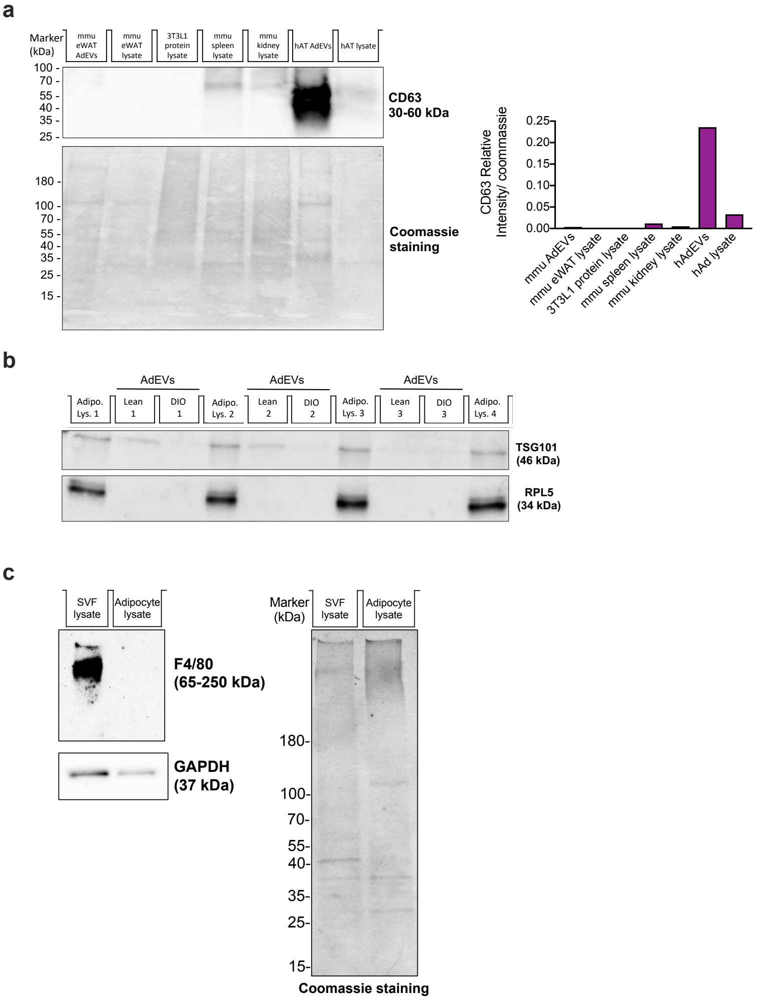

Western blot-based analysis of EVs and contamination markers

(**a**) Western blot analysis showing the presence of CD63 in the mouse spleen and kidney protein lysates as well as in human (h) derived AdEVs and protein lysate but no clear band for the murine (mmu) epididymal white adipose tissue (eWAT) AdEVs, eWAT lysate and 3T3-L1-derived EVs. Coomassie staining serves as a loading control. (**b**) Equal number of SEC isolated AdEVs from murine adipose tissue (2,3* 10^9 particles referring to 15 µg of protein) and 15 µg protein of the corresponding tissue lysate (n=3 biological replicates) were loaded on a 12% SDS-PAGE gel and transferred onto a PVDF membrane. Western Blot analysis showed the expression of the EV marker TSG101 in both lean and DIO AdEVs (upper lane). After stripping and re-probing of the same membrane, the absence of RPL-5 in all EV replicates but its presence in the control adipocyte lysates (lower lane) could be demonstrated. (**c**) 10 µg of total protein of the adipocyte fraction and stromal vascular fraction (SVF) of the eWAT lysate were loaded on a 4-20% gradient gel and transferred onto a PVDF membrane. Western blot analysis showed the expression of F4/80, a macrophage marker, in SVF protein lysate but no band was observed in the adipocyte protein lysate. GAPDH and Coomassie staining serve as loading controls, indicating equal amounts of protein in both adipocyte and SVF samples. Murine tissue samples were derived from 3 months old male C57BL/6J mice and human samples were taken from the study cohort described in supplementary table 3.

Supplementary Figure 4:

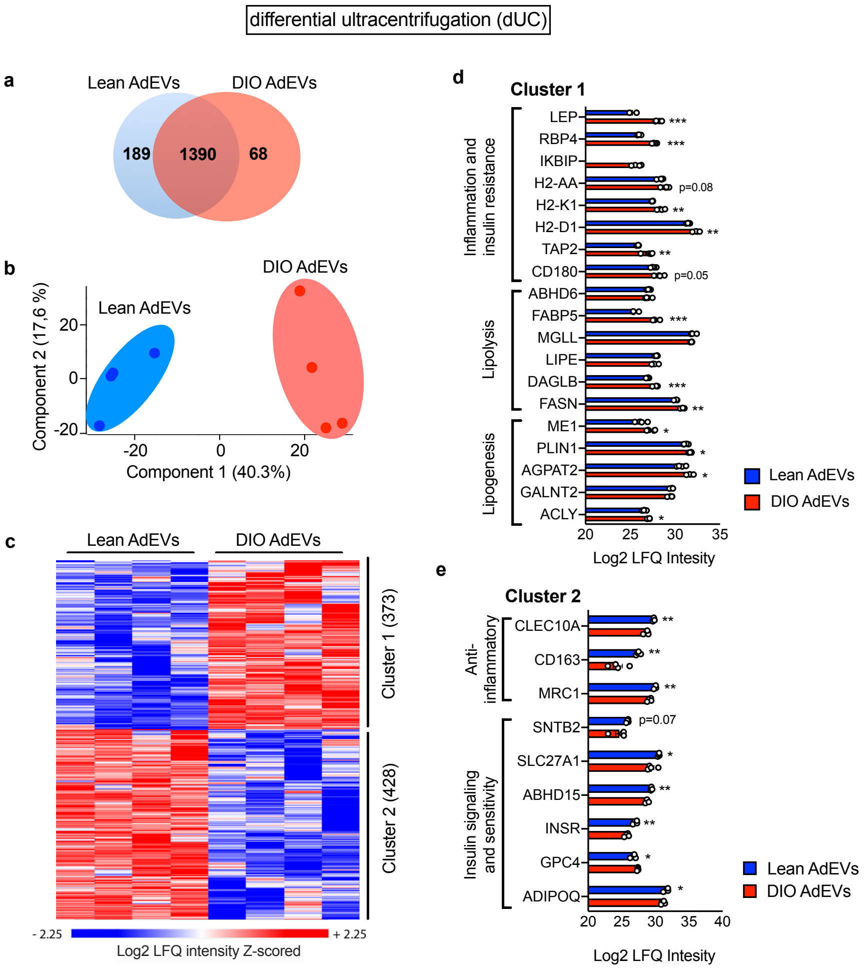

Mass spectrometry of dUC isolated AdEVs and their impact on the MIN6 proteome.

AdEVs were isolated via dUC from male 6-8 months old C57BL/6J mice subjected to 4-6 months of HFD or chow (visceral fat pads of 5 DIO and 10 lean mice were pooled for one biological replicate) and subjected to LC-MS/MS-based proteomics. (**a**) Venn Diagram and (**b**) principal component analysis (PCA) of proteins identified in lean and/or DIO AdEVs (n=4 biological replicates). (**c**) Heatmap of z-scored protein intensities for all uncovered proteins (ANOVA, FDR <0.01), and (**d-e**) average Log2LFQ intensities of selected proteins with different abundance in lean and DIO AdEVs. (**d**) Cluster 1: proteins involved in lipogenesis, lipolysis/FAO and inflammation and insulin resistance; (**e**) Cluster 2: proteins involved in anti-inflammatory responses, insulin signaling and sensitivity. Data are presented as mean values ± SEM (**d-e**). Significance was determined by two-samples two-sided t-test with permutation-based FDR set to 0.1 (**d-e**), *p<0.05, **p<0.01, ***p<0.001. Exact P values for Log2 LFQ Intensity differences between lean AdEVs vs DIO AdEVs are (**d**) P=0.000805518 (LEP), P=0.0000288809 (RBP4), P=0.0812971 (H2-AA), P=0.00238065 (H2-K1), P=0.00306158 (H2-D1), P=0.00705518 (TAP2), P=0.0539845 (CD180), P=0.00095055 (DAGLB), P=0.00119265 (FASN), P=0.0264985 (ME1), P=0.0129584 (PLIN1), P=0.0116015 (AGPAT2), P=0.0117036 (ACLY) and (**e**) P=0.0474604 (ADIPOQ), P=0.0186942 (GPC4), P=0.001219 (INSR), P=0.00115398 (ABHD15), P=0.023154 (SLC27A1), P=0.0766462 (SNTB2), P=0.00184708 (MRC1), P=0.0044646 (CD163), P=0.00133298 (CLEC10a).

Supplementary Figure 5:

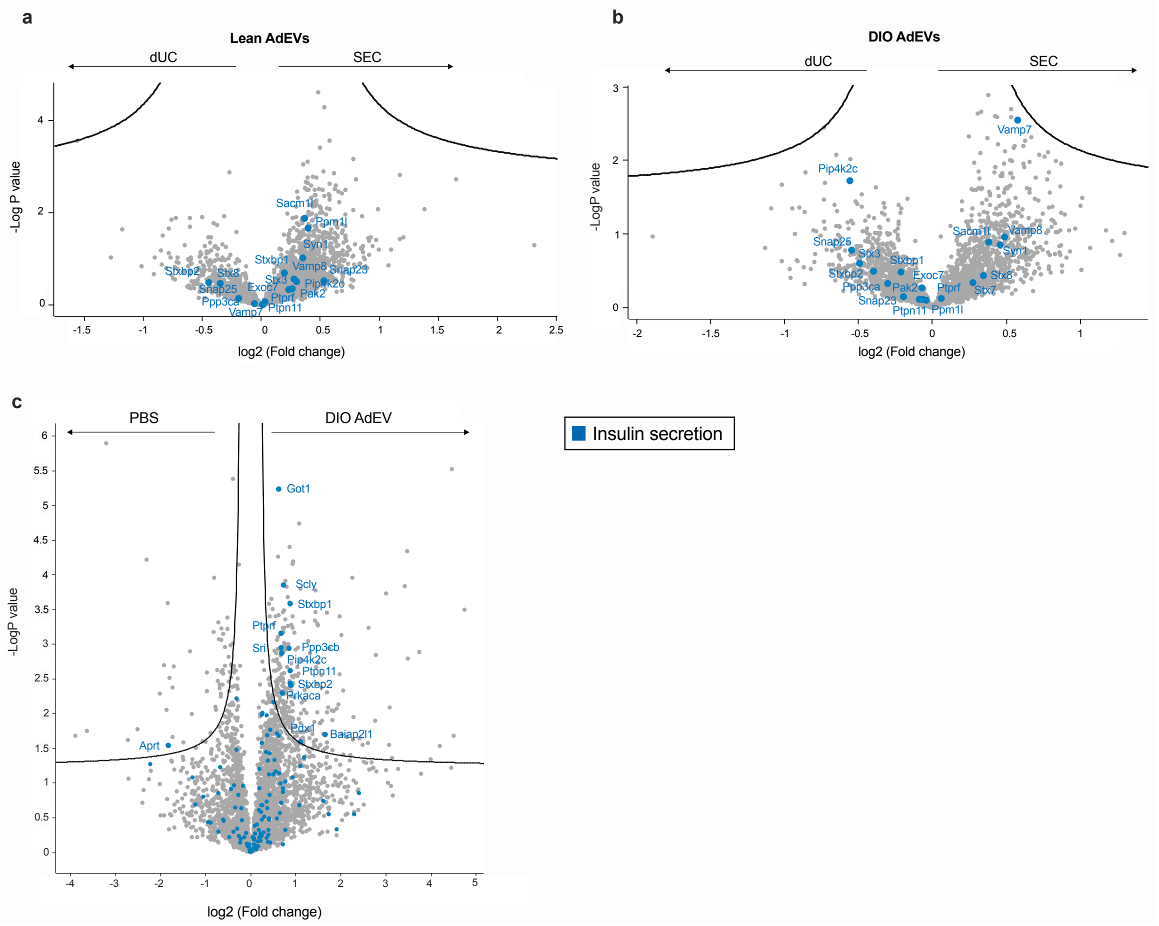

Mass spectrometry of SILAC labeled MIN6 cells after treatment with SEC or dUC isolated AdEVs

MIN6 cells were SILAC labeled over 8 passages with $Arg_{10}Lys_8$ amino acids and later treated for 6 hours with only PBS (Vehicle) or dUC- or SEC-isolated AdEVs (n=4 biologically independent samples) and analyzed by mass spectrometry. Each sample was pooled from visceral fat pads of 5 DIO or 10 lean male 6-8 months old C57BL/6J mice subjected to 4-6 months of HFD or chow. After background subtraction to correct for possible unlabeled host cell proteins, volcano plots, representing the fold change difference of the protein abundance of SEC-isolated lean (**a**) and obese (**b**) AdEVs versus the dUC-isolated AdEVs (n=4 biologically independent AdEV replicates), were generated. Proteins labeled in blue represent proteins involved in the insulin secretion pathway. (**c**) Volcano plot representing the fold change difference of the protein abundance of MIN6 cells treated with dUC isolated DIO AdEVs versus PBS control. Significance was determined by t-test with a false discovery rate (FDR) of 0.1.

Supplementary Figure 6:

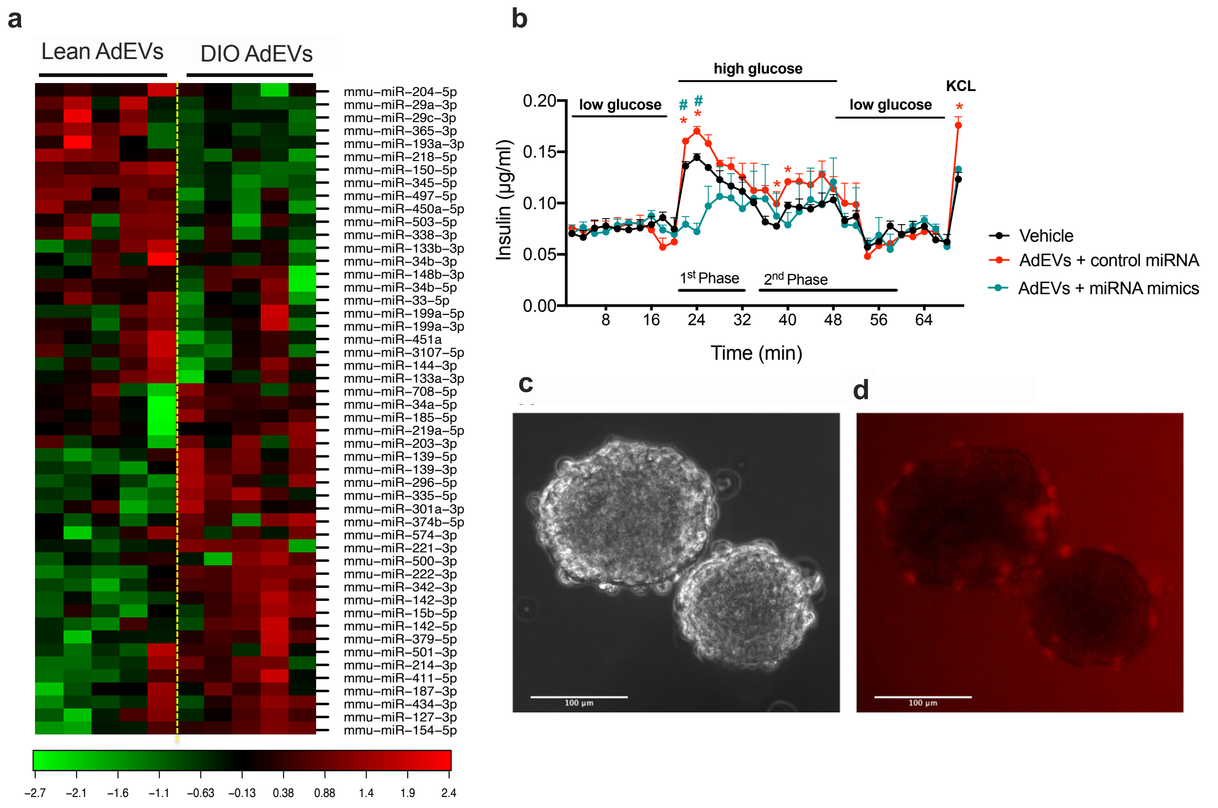

MicroRNA content of AdEVs and impact on insulin secretion in pancreatic islets.

Each AdEV sample was isolated from pooled visceral fat pads of 5 DIO or 10 lean male 6-8 months old C57BL/6J mice subjected to 4-6 months of HFD or chow. (**a**) Heatmap with two-way hierarchical clustering of microRNAs and samples. (**b**) Dynamic islet perfusion assay in murine islets transfected with a mixture of miR-29a-3p, miR-200a-3p, miR-218-5p and miR-322-5p mimics, or with non-functional control miRNA (n=3 biologically independent samples). (**c-d**) Representative phase contrast microscopy (**c**) and fluorescent (**d**) images of primary murine pancreatic islets after up to 18 hours of exposure with Texas Red end-labeled siRNA, demonstrating no uptake of native siRNAs into the islets. Scale bars represent 100 µm. Data are presented as mean values ± SEM. Asterisks indicate significance (*P<0.05) between the vehicle group and the AdEV+control miRNA group; Hash symbol indicates significance ($^{\#}$P<0.05) between the AdEVs + control miRNA group and the AdEV + miRNA mimics group. Exact P values for treatment effects are (**b**) P=0.0183 (vehicle vs. AdEVs + miRNA mimics, time point 22 min), P=0.0111 (AdEVs + miRNA mimics vs. AdEVs + control miRNA, time point 22min), P=0.0408 (vehicle vs. AdEVs + miRNA mimics, time point 24min), P=0.0163 (AdEVs + miRNA mimics vs. AdEVs + control miRNA, time point 24min), P=0.0395 (vehicle vs. AdEVs + control miRNA, time point 36min), P=0.0445 (vehicle vs. AdEVs + control miRNA, time point 40min), P=0.0258 (vehicle vs. AdEVs+ control miRNA, time point 70min).

Supplementary Figure 7:

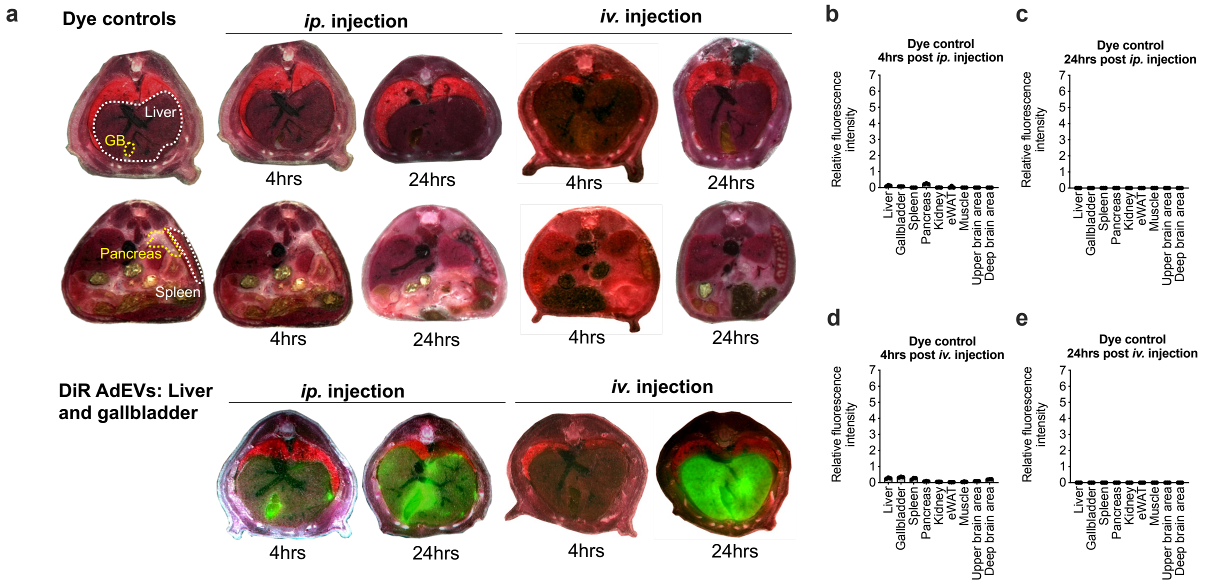

Tissue specific uptake of iv. injected AdEVs in mice

(**a**) Representative cross-sections of multiscale and multispectral images of whole cryo-sliced male lean C57BL/6J mice (11 to 16 weeks of age) along the axial planes after color and fluorescence image fusion. Liver and gall bladder (GB, upper lane) or spleen and pancreas (middle lane) 4 and 24 hours after intraperitoneal (ip.) or intravenous (iv.) injection with the DiR dye control. Lower lane: Livers of DiR-AdEV-injected mice 4 and 24 hours post ip. or iv. injection. (**b-d**) Relative quantification of fluorescence intensities 4 and 24 hours after ip. (**b-c**) and iv. (**d-e**) injection of dye control. Three representative slices from animals (n=2 mice) were selected. Each image stack was normalized against the average maximum fluorescence intensity of the stack corresponding to the controls. Regions of interest were defined to cover the complete cross-section of the organ from which the relative fluorescence intensities were calculated. Data are presented as mean values ± SEM.

Supplementary Figure 8:

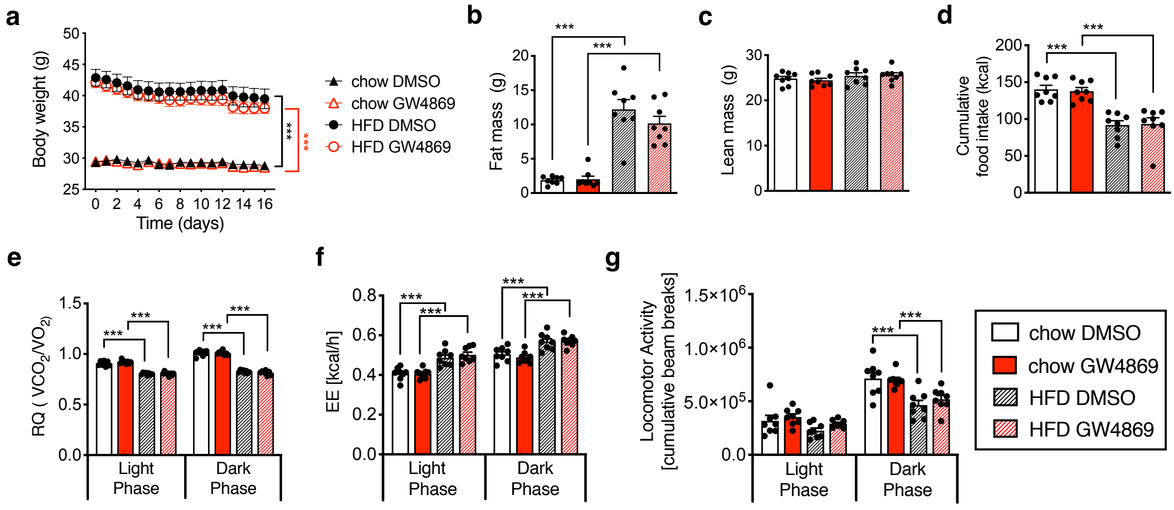

Metabolic phenotyping of GW4869-treated mice

Male chow fed lean and DIO C57BL/6J mice (28 weeks of age, 16 weeks of HFD feeding, resulting in a significant BW difference of 15.5 g) were daily injected with vehicle or 1 mg/kg of the small molecule inhibitor GW4869 for 16 days. We observed unaltered body weight (**a**), fat and lean mass (**b-c**) and cumulative food intake (**d**). High-fat diet feeding but not inhibitor treatment resulted in the expected decrease of the respiratory quotient (RQ, **e**). Similarly, energy expenditure (EE, **f**) and locomotor activity (**g**) were only affected by diet and dark-light shifts, but not by GW4869 treatment. n=8 mice. Data are presented as mean values ± SEM. Asterisks indicate *P<0.05, **P<0.01, ***P<0.001. Significance was determined by two-way ANOVA followed by Tukey's multiple comparisons test. Exact P values are (**a-g**) all P<0.0001 (chow DMSO vs. HFD DMSO and chow GW4869 vs. HFD GW4869).

SUPPLEMENTARY TABLES

Supplementary Table 1: Significant abundance of 19 miRNAs in AdEVs isolated from lean and diet-induced obese (DIO) mice as indicated by the fold change (FC) between the groups. The last two columns show the p-value from the t-test and the Benjamini-Hochberg adjusted p-value.

miRNA	FC (DIO / lean)	t-test p-value	BH adj. p-value
mmu-miR-101a-3p	-1,6715	0.0001	0.0137
mmu-miR-143-3p	-1.6183	0.0002	0.0137
mmu-miR-222-3p	4.0947	0.0003	0.0137
mmu-miR-150-5p	-2.4025	0.0003	0.0137
mmu-miR-345-5p	-2.5334	0.0003	0.0137
mmu-miR-27b-3p	1.6139	0.0006	0.0183
mmu-miR-139-3p	5.6558	0.0007	0.0183
mmu-miR-181a-5p	-1.6744	0.0007	0.0183
mmu-miR-322-5p	-1.7413	0.0009	0.0189
mmu-miR-142-3p	2.4302	0.0009	0.0189
mmu-miR-126a-3p	-1.7111	0.0014	0.0262
mmu-miR-24-3p	1.5757	0.0016	0.0269
mmu-miR-376a-3p	3.3743	0.0029	0.0440
mmu-miR-342-3p	4.3555	0.0032	0.0440
mmu-miR-425-5p	-1.4712	0.0034	0.0440
mmu-miR-146b-5p	1.7666	0.0036	0.0440
mmu-miR-29b-3p	-1.4181	0.0038	0.0440
mmu-miR-18a-5p	1.7924	0.0038	0.0440
mmu-miR-450a-5p	-2.6134	0.0045	0.0487

Supplementary Table 2: MicroRNAs detected in AdEVs isolated from eWAT adipocyte explants of lean and diet-induced obese (DIO) mice. MicroRNAs are classified depending on their reported functions and mechanisms in glucose-stimulated insulin secretion and insulin gene transcription, their respective fold-changes between lean and DIO mice, and the respective p-values from t-tests. *p < 0.05, **p < 0.01, ***p < 0.001.

Function	miRNA	Mechanism	References	FC (DIO / lean)	t-test p-value
Fuel uptake and Ca2+ influx	miR-29b	Repressor of Monocarboxylate Transporter 1	11	-1.418	0.004**
	miR-145a	Repressor of ABCA1 expression	12	-1.445	0.008**
Exocytosis and insulin secretion	miR-9	Negative regulator of insulin secretion by repression of Stxbp1	13	-1.052	0.946
	miR-21a	Negative regulator of insulin secretion	14	-1.085	0.565
	miR-29a	Negative regulator of insulin secretion by repression of Stx1a	15, 16	-1.730	0.045*
	miR-34a	Negative regulator of insulin secretion	17	1.478	0.198
	miR-130a	Negative regulator of insulin secretion	18	-1.017	0.897
	miR146a	Negative regulator of insulin secretion	17	1.072	0.690
	miR-200a	Negative regulator of insulin secretion	18	-3.725	0.066
	miR-218	Negative regulator of insulin secretion by repression of Stxbp1	19	-2.53	0.0061**
	miR-322	Negative regulator of insulin secretion by repression of Stxbp1	19	-1.741	0.001***
	miR-335	Negative regulator of insulin secretion	20	4.192	0.007**
	miR-375	Negative regulator of insulin secretion	21	-3.158	0.120
	miR-410	Negative regulator of insulin secretion	18	1.210	0.550
Insulin gene regulation	miR-15a	Positive regulation of insulin synthesis by targeting UCP2	22	-1.128	0.335
	miR-19b	Downregulation of insulin 1 through targeting transcription factor NeuroD1	23	-1.248	0.053
	miR-24	Positive regulator of insulin transcription	24	1.576	0.002**
	miR-25	Positive regulator of insulin transcription	25	-1.132	0.369
	miR-26	Upregulation of insulin transcription	24	-1.594	0.005**
	miR-30d	Upregulation of insulin transcription	26	1.047	0.791
	miR-148	Positive regulator of insulin transcription	24	1.106	0.535
	miR-204	Downregulation of insulin transcription by targeting MafA	27	-1.721	0.0838

Supplementary Table 3: Clinical data from human liposuction patients (mean age (years) ± SD: 37.8 ± 7.2), with 5 being overweight individuals and 6 falling within the healthy weight range. Samples indicated with a star symbol are from the same patient from two individual surgeries and were considered as individual samples.

Sample ID	Gender	Body mass index [kg/m^2]
1- p568	female	27
2- p574	female	28,6
3- p575	female	19,6
4- p576	female	27,8
5- p577	female	27,7
6- p578	female	26,3
7- p579	female	19,3
8- p581	female	21,1
9*- p582	female	21,5
10*- p583	female	21,5
11- p587	female	22,8

Un-cropped Blots for Supplementary Figure 3a

Un-cropped blot: Exposure time: 3 mins

Mouse anti-CD63. MW: 30-60 kDa

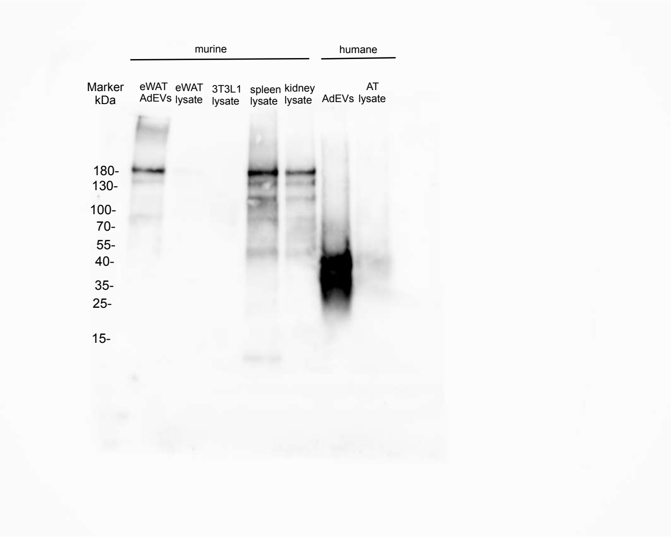

Un-cropped blot for marker detection_colorimetric image

Page Ruler^{TM} Prestained protein ladder, 10to 180 kDa, Thermofisher, Cat.No: 26616

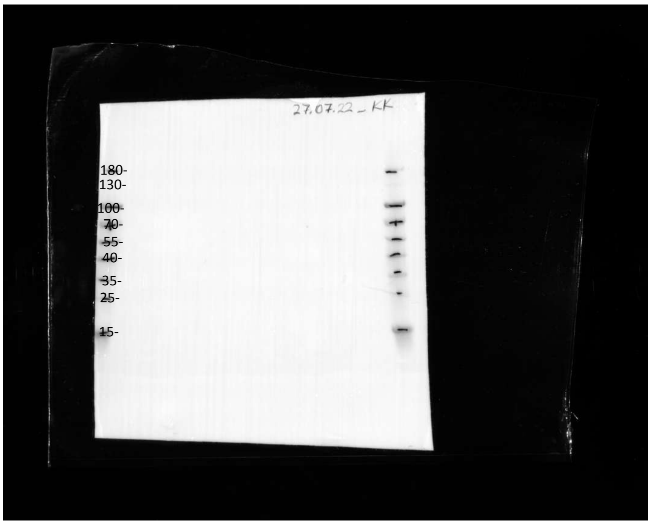

Un-cropped coomassie blot

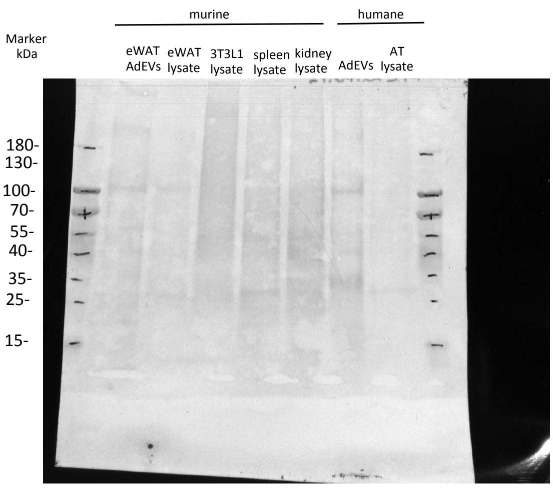

Resulting Supplementary Figure S3A

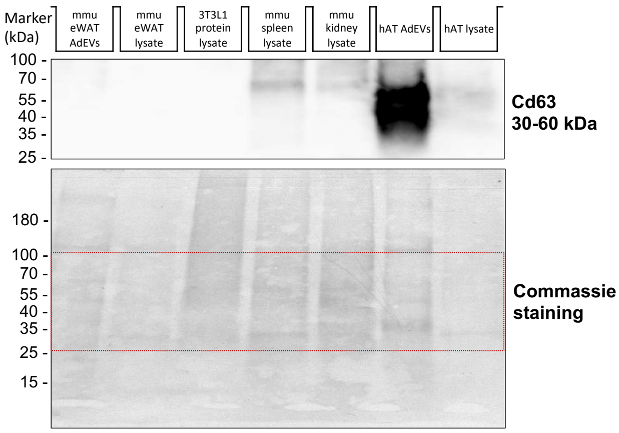

Un-cropped Blots for Supplementary Figure 3b

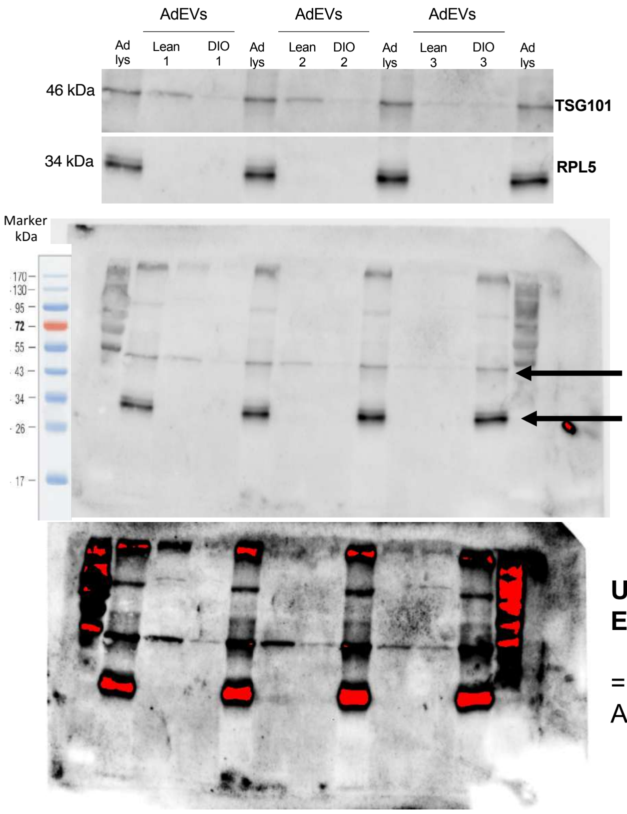

Un-cropped blot:
Exposure time: 2 mins

Rabbit Anti-TSG. MW: 46 kDa

Rabbit Anti-RPL5. MW: 34 kDa

Un-cropped blot:
Exposure time: 20 mins

=> Absence of RPL5 in AdEV samples

Un-cropped Blots for Supplementary Figure 3c

Un-cropped blot: Exposure time: 4 mins

Rabbit anti-F4/80. MW: 65-250 kDa

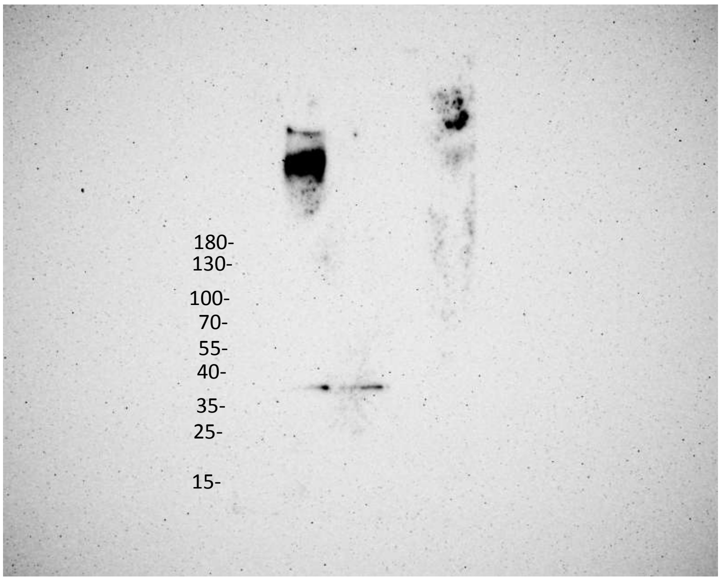

Un-cropped blot for marker detection. Colorimetric image

Page Ruler^{TM} Prestained protein ladder, 10to 180 kDa, Thermofisher, Cat.Nr.: 26616

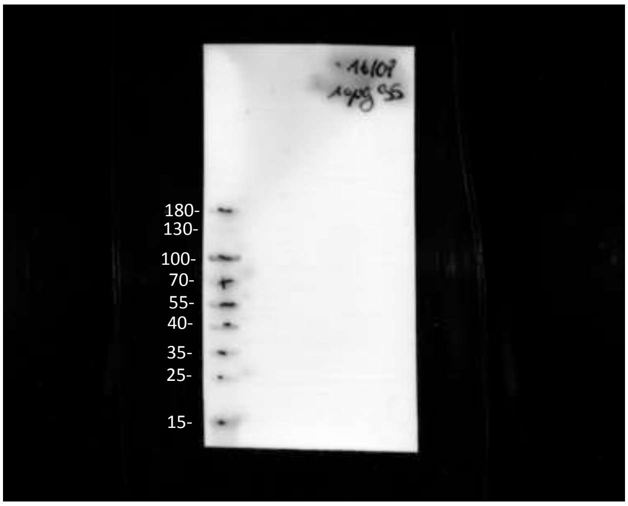

Un-cropped blot: Exposure time: 5 secs

Mouse anti-GAPDH. (Millipore, 1/3000, CB1001) MW: 37 kDa

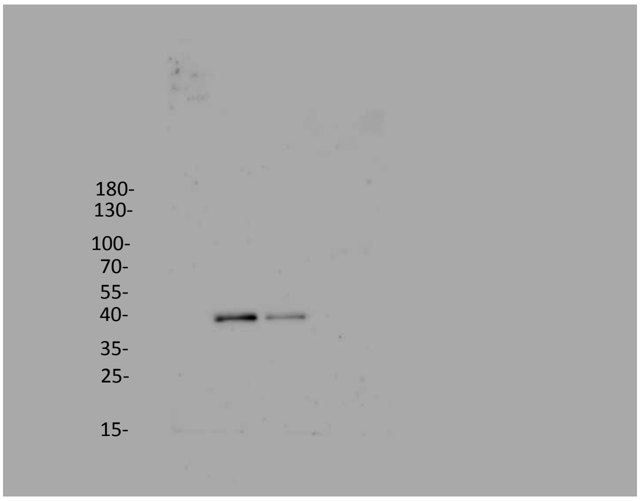

Un-cropped coomassie blot

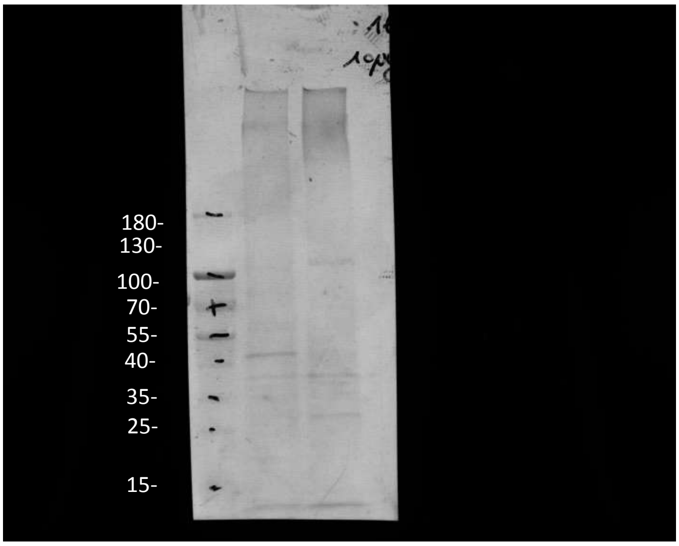

SUPPLEMENTARY REFERENCES

1. Mastronarde DN. Automated electron microscope tomography using robust prediction of specimen movements. *J Struct Biol* **152**, 36-51 (2005).

2. Zheng SQ, Palovcak E, Armache JP, Verba KA, Cheng Y, Agard DA. MotionCor2: anisotropic correction of beam-induced motion for improved cryo-electron microscopy. *Nat Methods* **14**, 331-332 (2017).

3. Schindelin J*, et al.* Fiji: an open-source platform for biological-image analysis. *Nat Methods* **9**, 676-682 (2012).

4. Kulak NA, Pichler G, Paron I, Nagaraj N, Mann M. Minimal, encapsulated proteomic-sample processing applied to copy-number estimation in eukaryotic cells. *Nat Methods* **11**, 319-324 (2014).

5. Humphrey SJ, Karayel O, James DE, Mann M. High-throughput and high-sensitivity phosphoproteomics with the EasyPhos platform. *Nat Protoc* **13**, 1897-1916 (2018).

6. Merl-Pham J*, et al.* Quantitative proteomic profiling of extracellular matrix and site-specific collagen post-translational modifications in an in vitro model of lung fibrosis. *Matrix Biol Plus* **1**, 100005 (2019).

7. Tyanova S*, et al.* The Perseus computational platform for comprehensive analysis of (prote)omics data. *Nat Methods* **13**, 731-740 (2016).

8. Tyanova S, Cox J. Perseus: A Bioinformatics Platform for Integrative Analysis of Proteomics Data in Cancer Research. *Methods Mol Biol* **1711**, 133-148 (2018).

9. Mita M*, et al.* Green Fluorescent Protein-Based Glucose Indicators Report Glucose Dynamics in Living Cells. *Anal Chem* **91**, 4821-4830 (2019).

10. Barapatre N*, et al.* Quantitative detection of drug dose and spatial distribution in the lung revealed by Cryoslicing Imaging. *J Pharm Biomed Anal* **102**, 129-136 (2015).

11. Pullen TJ, da Silva Xavier G, Kelsey G, Rutter GA. miR-29a and miR-29b contribute to pancreatic beta-cell-specific silencing of monocarboxylate transporter 1 (Mct1). *Mol Cell Biol* **31**, 3182-3194 (2011).

12. Kang MH*, et al.* Regulation of ABCA1 protein expression and function in hepatic and pancreatic islet cells by miR-145. *Arterioscler Thromb Vasc Biol* **33**, 2724-2732 (2013).

13. Hu D, Wang Y, Zhang H, Kong D. Identification of miR-9 as a negative factor of insulin secretion from beta cells. *J Physiol Biochem* **74**, 291-299 (2018).

14. Sims EK, Lakhter AJ, Anderson-Baucum E, Kono T, Tong X, Evans-Molina C. MicroRNA 21 targets BCL2 mRNA to increase apoptosis in rat and human beta cells. *Diabetologia* **60**, 1057-1065 (2017).

15. Bagge A, Dahmcke CM, Dalgaard LT. Syntaxin-1a is a direct target of miR-29a in insulin-producing beta-cells. *Horm Metab Res* **45**, 463-466 (2013).

16. Duan J*, et al.* miR-29a Negatively Affects Glucose-Stimulated Insulin Secretion and MIN6 Cell Proliferation via Cdc42/beta-Catenin Signaling. *Int J Endocrinol* **2019**, 5219782 (2019).

17. Roggli E*, et al.* Involvement of microRNAs in the cytotoxic effects exerted by proinflammatory cytokines on pancreatic beta-cells. *Diabetes* **59**, 978-986 (2010).

18. Hennessy E, Clynes M, Jeppesen PB, O'Driscoll L. Identification of microRNAs with a role in glucose stimulated insulin secretion by expression profiling of MIN6 cells. *Biochem Biophys Res Commun* **396**, 457-462 (2010).

19. Lang H*, et al.* Characterization of miR-218/322-Stxbp1 pathway in the process of insulin secretion. *J Mol Endocrinol* **54**, 65-73 (2015).

20. Salunkhe VA*, et al.* MiR-335 overexpression impairs insulin secretion through defective priming of insulin vesicles. *Physiol Rep* **5**, (2017).

21. Poy MN*, et al.* A pancreatic islet-specific microRNA regulates insulin secretion. *Nature* **432**, 226-230 (2004).

22. Sun LL, Jiang BG, Li WT, Zou JJ, Shi YQ, Liu ZM. MicroRNA-15a positively regulates insulin synthesis by inhibiting uncoupling protein-2 expression. *Diabetes Res Clin Pract* **91**, 94-100 (2011).

23. Zhang ZW*, et al.* MicroRNA-19b downregulates insulin 1 through targeting transcription factor NeuroD1. *FEBS Lett* **585**, 2592-2598 (2011).

24. Melkman-Zehavi T*, et al.* miRNAs control insulin content in pancreatic beta-cells via downregulation of transcriptional repressors. *EMBO J* **30**, 835-845 (2011).

25. Setyowati Karolina D, Sepramaniam S, Tan HZ, Armugam A, Jeyaseelan K. miR-25 and miR-92a regulate insulin I biosynthesis in rats. *RNA Biol* **10**, 1365-1378 (2013).

26. Zhao X, Mohan R, Ozcan S, Tang X. MicroRNA-30d induces insulin transcription factor MafA and insulin production by targeting mitogen-activated protein 4 kinase 4 (MAP4K4) in pancreatic beta-cells. *J Biol Chem* **287**, 31155-31164 (2012).

27. Xu G, Chen J, Jing G, Shalev A. Thioredoxin-interacting protein regulates insulin transcription through microRNA-204. *Nat Med* **19**, 1141-1146 (2013).
