# Supplementary material for: Adipocyte-derived extracellular vesicles increase insulin secretion through transport of insulinotropic protein cargo
Source: Nat Commun. 2023 Feb 9;14:709. doi: 10.1038/s41467-023-36148-1 (PMC9911726; doi:10.1038/s41467-023-36148-1)
Supplement: Supplementary file 2 — Reporting Summary [file 41467_2023_36148_MOESM2_ESM.pdf]

nature portfolio

Corresponding author(s): Kerstin Stemmer

Last updated by author(s): 19.12.2022

Reporting Summary

Nature Portfolio wishes to improve the reproducibility of the work that we publish. This form provides structure for consistency and transparency in reporting. For further information on Nature Portfolio policies, see our Editorial Policies and the Editorial Policy Checklist.

Statistics

For all statistical analyses, confirm that the following items are present in the figure legend, table legend, main text, or Methods section.

n/a	Confirmed	
☐	☒	The exact sample size (n) for each experimental group/condition, given as a discrete number and unit of measurement
☐	☒	A statement on whether measurements were taken from distinct samples or whether the same sample was measured repeatedly
☐	☒	The statistical test(s) used AND whether they are one- or two-sided<br>*Only common tests should be described solely by name; describe more complex techniques in the Methods section.*
☐	☒	A description of all covariates tested
☐	☒	A description of any assumptions or corrections, such as tests of normality and adjustment for multiple comparisons
☐	☒	A full description of the statistical parameters including central tendency (e.g. means) or other basic estimates (e.g. regression coefficient) AND variation (e.g. standard deviation) or associated estimates of uncertainty (e.g. confidence intervals)
☐	☒	For null hypothesis testing, the test statistic (e.g. F, t, r) with confidence intervals, effect sizes, degrees of freedom and P value noted<br>*Give P values as exact values whenever suitable.*
☒	☐	For Bayesian analysis, information on the choice of priors and Markov chain Monte Carlo settings
☒	☐	For hierarchical and complex designs, identification of the appropriate level for tests and full reporting of outcomes
☒	☐	Estimates of effect sizes (e.g. Cohen's d, Pearson's r), indicating how they were calculated

Our web collection on statistics for biologists contains articles on many of the points above.

Software and code

Policy information about availability of computer code

Data collection	Videos from nanoparticle tracing analysis (NTA) were analyzed by the inbuilt ZetaView Software 8.05.11 SP1. Transmission electron cryomicroscopy (Cryo-TEM) images were recorded with SerialEM software on a Tecnai G2 Polara transmission electron microscope (FEI, Thermo Fisher Scientific). The frame stacks were subjected to alignment and dose filtering using MotionCor2. Raw proteomics data were processed with MaxQuant version 1.6.14.0. Whole mouse Cryoslicing Imaging was performed with a conventional cryo-microtome (CM 1950, Leica Microsystems GmbH, Wetzlar, Germany) equipped with an imaging unit capable of recording red, green and blue (RGB) color images and fluorescence images at multiple wavelengths using a 24-85 mm zoom lens (Nikkor, Tokyo, Japan) and a NIR sensitive camera (Luca R, Andor Technologies, Belfast, UK) and excitation and emission filter wheels. A detailed description of the data and sample collection can be found in the methods section and in the supplemental material of the manuscript. Confocal microscopy images were taken with a LEICA TCS SP5 microscope and processed with the Leica Application Suite Advanced Fluorescence (LAS AF) software (version: 2.7.3.9723).
Data analysis	For proteome analyses, searches were performed against the mouse or human Uniprot FASTA database (2019). Perseus (version 1.6.14.0) was used for bioinformatics analysis. UniProtKB, Gene Ontology (GO), and the Kyoto Encyclopedia of Genes and Genomes (KEGG) annotations were included in the analysis. Statistical analysis were made using Graphpad Prism (version 8.0). A detailed description of the analysis can be found in the methods section and in the supplemental material of the manuscript.

For manuscripts utilizing custom algorithms or software that are central to the research but not yet described in published literature, software must be made available to editors and reviewers. We strongly encourage code deposition in a community repository (e.g. GitHub). See the Nature Portfolio guidelines for submitting code & software for further information.

Data

Policy information about availability of data

All manuscripts must include a data availability statement. This statement should provide the following information, where applicable:

- Accession codes, unique identifiers, or web links for publicly available datasets
- A description of any restrictions on data availability
- For clinical datasets or third party data, please ensure that the statement adheres to our policy

Proteomics data have been deposited to the ProteomeXchange Consortium via the PRIDE partner repository (https://www.ebi.ac.uk/pride/) with the dataset identifier PXD037809. Source data are provided with this paper

Human research participants

Policy information about studies involving human research participants and Sex and Gender in Research.

Reporting on sex and gender	Our study only included female adipose tissue donors. Sexes were self-reported.
Population characteristics	Random female liposuction patients, with body mass indexes ranging from 19 to 29. Ages ranged from 24 to 47 years.
Recruitment	No active recruitment. All human adipose tissue donors gave written informed consent.
Ethics oversight	The study protocol was approved by the ethics committee of the Technical University of Munich.

Note that full information on the approval of the study protocol must also be provided in the manuscript.

Field-specific reporting

Please select the one below that is the best fit for your research. If you are not sure, read the appropriate sections before making your selection.

☒ Life sciences ☐ Behavioural & social sciences ☐ Ecological, evolutionary & environmental sciences

For a reference copy of the document with all sections, see nature.com/documents/nr-reporting-summary-flat.pdf

Life sciences study design

All studies must disclose on these points even when the disclosure is negative.

Sample size	Sample size for in vivo experiments (glucose tolerance tests) is based on power analyses and previous experiments to obtain significance and reproducibility (Stemmer et al., Diabetologia 2015; Quarta, Stemmer, Novikoff, et al., Nature Metabolism 2022)<br>For in vitro experiments, sample sizes were based on empirical data from pilot experiments. The experiments followed common standards employing three or more biological replicates. Sample sizes are given in the figure captions.
Data exclusions	In Figure 1n Grubb's test excluded 3 outliers that were then excluded from further analyses. The respective values are and the Grubb's test results are shown in the source file. Two samples were excluded from the proteomics dataset in Figure 3. The samples had insufficiently low counts of valid values compared to the corresponing replicate samples indicative for reduced data quality. Accordingly both samples were identified as ouliers in the PCA and excluded from all further analyses.
Replication	Validity of the glucoregulatory effects of AdEVs were replicated in vivo in several independent experiments e.g. by different time-points and different routes of injection. In vitro studies were performed with 3-4 biological replicates. Validity of proteomics results in SEC-isolated AdEV have been confirmed in similar experiments using dUC-isolated AdEVs.
Randomization	Lean mice and DIO mice were randomized for AdEV exposure experiments based on body weights, with vehicle and treatment groups being matched for body weight. No other covariates have been considered. Samples from random liposuction patients have been collected over a time period of two years.
Blinding	The daily scoring of experimental mice for a general health assessment is mandated by our government, requires specific scoring for the respective animal identities and their treatment groups (for AdEV isolations and studies), and thus prohibits full blinding of all participating researchers. Nonetheless, for our in vivo experiments (GTTs) most handling investigators were blinded to the treatment condition. In vitro studies were conducted by single investigators and were hence not blinded. For the analysis of microscopic images (cryo-TEM and in vivo cryo slicing) handling investigators were blinded to the treatment condition.

March 2021

Reporting for specific materials, systems and methods

We require information from authors about some types of materials, experimental systems and methods used in many studies. Here, indicate whether each material, system or method listed is relevant to your study. If you are not sure if a list item applies to your research, read the appropriate section before selecting a response.

Materials & experimental systems

n/a	Involved in the study
☐	☒ Antibodies
☐	☒ Eukaryotic cell lines
☒	☐ Palaeontology and archaeology
☐	☒ Animals and other organisms
☐	☒ Clinical data
☒	☐ Dual use research of concern

Methods

n/a	Involved in the study
☒	☐ ChIP-seq
☒	☐ Flow cytometry
☒	☐ MRI-based neuroimaging

Antibodies

Antibodies used

TSG-101 (Sigma Aldrich, 1/1000, HPA006161)
RPL-5 (Cell Signaling, 1/1000, 14568)
CD63 (Santa Cruz, 1/1000, sc-5275)
F4/80 (Cell Signaling, 1/1000, 70076T)
GAPDH (Millipore, 1/3000, CB1001)
IgG-HRP (Santa Cruz, sc-2005)
goat anti-rabbit (Santa Cruz, sc-2030)
Insulin (Thermo Fisher Scientific, PA1-26938)
Donkey anti-guinea pig Jackson ImmunoResearch Europe, UK706-545-148

Validation

All antibodies were validated for the species and application as indicated by the manufacturer (see manufacturer's website). Specific inormation is given below.

TSG-101 (Sigma Aldrich, HPA006161)
The TSG-101 (Sigma Aldrich, HPA006161) belongs to the group of Prestige Antibodies®, which are highly characterized and extensively validated antibodies with the added benefit of all available characterization data for each target being accessible via the Human Protein Atlas portal linked just below the product name at the top of this page. The uniqueness and low cross-reactivity of the Prestige Antibodies® to other proteins are due to a thorough selection of antigen regions, affinity purification, and stringent selection. Every Prestige Antibody is tested in the following ways: 1. IHC tissue array of 44 normal human tissues and 20 of the most common cancer type tissues. 2. Protein array of 364 human recombinant protein fragments.

RPL-5 (Cell Signaling, 14568)
RPL-5 (Cell Signaling #14568) is a polyclonal antibody validated and recommended by the manufacturer for use in western blot. The antibody is specified to react with human, mouse, rat and monkey RPL-5. The antibody was validated in a western blot analysis with extracts from various cell lines (human, mouse, rat, monkey).

CD63 (Santa Cruz, sc-5275)
CD63 antibody (Santa Cruz, #sc-5275, clone MX-49.129.5) is a mouse monoclonal IgG1 κ CD63 antibody, cited in 460 publications. It is validated and recommended by the manufacturer for detection of CD63 of mouse, rat and human origin by western blotting, immunoprecipitation, immunofluorescence, immunohistochemistry (including paraffin-embedded sections), flow cytometry and solid phase ELISA.

F4/80 (Cell Signaling, 70076T)
F4/80 XP® (Cell Signaling, #70076T, clone D2S9R) is a rabbit monoclonal IgG antibody against mouse F4/80 protein and was cited in 232 publications. The antibody is validated and recommended by the manufacturer for use in western blot, immunoprecipitation as well as immunohistochemistry (paraffin-embedded sections) and was tested in various murine cell lines (BaF3, Raw 264.7) and tissues (liver, spleen, small intestine). XP® monoclonal antibodies are high quality rabbit monoclonal antibodies exclusively available from Cell Signaling Technology, generated using a proprietary rabbit monoclonal method. XP® antibodies have met additional standards for exceptional performance, including high specificity and sensitivity in one or more key applications.

GAPDH (Millipore, CB1001)
GAPDH (Millipore, #CB1001) is recommended by the manufacturer for immunohistochemistry, western blot and ELISA. The antibody is specified to react with canine, chicken, fish, frog, human, mouse, porcine, rabbit and rat GAPDH. The antibody was validated using immunohistochemical analysis of rat aortic smooth muscle cells showing staining of GAPDH. Further WB validation was performed on tissue extract from isolated human GAPDH, human heart, pig heart, goat heart, bovine heart, dog heart, mouse heart, rat heart, rabbit heart, and duck heart. The corresponding images are shown on the datasheet.

IgG-HRP (Santa Cruz, sc-2005)
Goat anti-mouse IgG-HRP (Santa Cruz, #sc-2005) is an affinity purified secondary antibody raised in goat against mouse IgG and conjugated to HRP (horseradish peroxidase). Goat anti-mouse IgG-HRP is recommended for detection of mouse IgG by western

blotting and ELISA. The antibody was validated using western blot analysis of β-Actin, GAPDH, c-Yes and α-actinin expression in different whole cell lysates. The corresponding images are shown on the datasheet.

goat anti-rabbit (Santa Cruz, sc-2030)
Goat anti-rabbit IgG-HRP (Santa Cruz, #sc-2030) is a CruzMarker™ compatible, affinity purified secondary antibody raised in goat against rabbit IgG and conjugated to HRP (horseradish peroxidase). It is recommended for detection of rabbit IgG by western blotting. The antibody was validated using western blot analysis of p19 ARF expression in C3H/10T1/2, NIH/3T3, 3T3-L1, MH-S, AMJ2-C8 and I-11.15 whole cell lysates. Further validation was performed on COL3A1 expression in Hs68, HeLa and An3 CA whole cell lysates. The corresponding images are shown on the datasheet.

Insulin (Thermo Fisher Scientific, PA1-26938)
Insulin (Thermo Fisher Scientific, #PA1-26938) is a polyclonal antibody recommended by the manufacturer for use in immunohistochemistry, immunocytochemistry and ELISA. The antibody is specified to react with human and mouse insulin. The antibody was validated using immunohistochemical analysis of paraffin-embedded human pancreas tissue showing staining of beta cells of the Langerhans's islets. The corresponding images are shown on the datasheet.

Donkey anti-guinea pig (Jackson ImmunoResearch Europe, UK706-545-148)
The Alexa Fluor 488-AffiniPure Donkey Anti-Guinea Pig IgG polyclonal antibody (Jackson ImmunoResearch Europe, #706-545-148) reacts with whole molecule guinea pig IgG based on immunoelectrophoresis and/or ELISA. It also reacts with the light chains of other guinea pig immunoglobulins. No antibody was detected against non-immunoglobulin serum proteins. The antibody has been tested by ELISA and/or solid phase adsorbed to ensure minimal cross-reaction with bovine, chicken, goat, syrian hamster, horse, human, mouse, rabbit, rat and sheep serum proteins, but it may cross-react with immunoglobulins from other species.
Whole IgG antibodies are isolated as intact molecules from antisera by immunoaffinity chromatography. They have an Fc portion and two antigen binding Fab portions joined together by disulfide bonds and therefore they are divalent. The average molecular weight is reported to be about 160 kDa. The whole IgG form of antibodies is suitable for the majority of immunodetection procedures and is the most cost effective.

Eukaryotic cell lines

Policy information about cell lines and Sex and Gender in Research

Cell line source(s)	MIN6 mouse clonal β-cells were provided by Prof. J. Miyazaki (Osaka University, Japan).
Authentication	The cell line was not authenticated.
Mycoplasma contamination	All cell lines were regularely tested negative for mycoplasma contamination.
Commonly misidentified lines (See ICLAC register)	No commonly misidentified cell lines were used in this manuscript

Animals and other research organisms

Policy information about studies involving animals; ARRIVE guidelines recommended for reporting animal research, and Sex and Gender in Research

Laboratory animals	Male C57BL/6J mice were purchased from Janvier Labs at an age of 6-8 weeks. Upon arrival, mice were group-housed and kept in a constant environment with the ambient temperature set to 22 ± 2°C with constant humidity (45 – 65%) and a 12h/12h light/dark cycle.
Wild animals	The study did not involve wild animals.
Reporting on sex	Only male mice were used for the study.
Field-collected samples	The study did not involve field-collected samples.
Ethics oversight	The animal experiments were performed in accordance with the European guidelines under permission of the local Animal Ethics Committee of the state of Bavaria, Germany.

Note that full information on the approval of the study protocol must also be provided in the manuscript.

Clinical data

Policy information about clinical studies
All manuscripts should comply with the ICMJE guidelines for publication of clinical research and a completed CONSORT checklist must be included with all submissions.

Clinical trial registration	The study does not include clinical trials or clinical trial associated data.
Study protocol	The study protocols were approved by the ethics committee of the Technical University of Munich (Study №: 5716/13 and 2361/09)
Data collection	White adipose tissue samples were collected at the time of liposuction surgery and stored overnight in medium at 4°C. The

Data collection	following morning, AdEVs were isolated and subsequently stored at -80°C for further analyses. Details are given in the supplementary material.
Outcomes	Adipose tissue EVs (from adipocytes and the stromal vascular fraction) were subjected to LC-MS analyses to assess proteome changes (primary endpoint).
